# The evolving study of political polarization

**DOI:** 10.12688/openreseurope.23252.1

**Published:** 2026-04-07

**Authors:** Emre Erdogan, Bernadette Nadya Jaworsky, Cláudia Álvares, Bojana Bodroža, Eglė Butkevičienė, Erlis Cela, Jérémy Dodeigne, Shahira S. Fahmy, Sarah Helena Schäfer, Graham Scott, Gonzalo Velasco Arias, Rocio Zamora Medina

**Affiliations:** 1 International Relations, Istanbul Bilgi University, Istanbul, Turkey; 2 Sociology, Masaryk University Faculty of Social Studies, Brno, South Moravian Region, 60200, Czech Republic; 3 Sociology, ISCTE-University Institute of Lisbon, Lisbon, Lisbon, Portugal; 4 Psychology, University of Novi Sad, Novi Sad, Serbia; 5 Kaunas University of Technology Faculty of Social Sciences Humanities and Arts, Kaunas, Kaunas County, Lithuania; 6 Western Balkans University, Tirana, Albania; 7 University of Namur, Namur, Walloon Region, Belgium; 8 Journalism and Mass Communications, The American University in Cairo School of Global Affairs and Public Policy, New Cairo City, Cairo Governorate, Egypt; 9 University of Vienna Department of Sociology, Vienna, Vienna, Austria; 10 University of the West of Scotland, Paisley, Scotland, UK; 11 University Carlos III of Madrid, Getafe, Community of Madrid, Spain; 12 University of Murcia Faculty of Communication and Information, Murcia, Region of Murcia, Spain

**Keywords:** polarization, methodology, epistemology

## Abstract

The study of political polarization has undergone significant conceptual and methodological evolution, progressing from early emphases on ideological divides toward a more complex understanding that incorporates affective, identity-based, digital, and cross-national dimensions. This shift reflects the growing recognition that polarization is not merely a divergence of policy preferences, but a multifaceted phenomenon shaped by emotional, social, technological, and epistemic processes. In this review, we provide an elaboration of the various ways to approach the study of political polarization. This task may be daunting, but a well-organized, comprehensive and interdisciplinary overview is surely necessary. We begin by elaborating different types of political polarization, moving from an early focus on ideological divides to multidimensional constructs. Then, we shift to an overview of key disciplinary concepts and theoretical approaches for examining political polarization. At the heart of this review, we elaborate the various methodologies and methods for studying polarization, including quantitative, computational/digital, experimental, and qualitative approaches. We also highlight the strengths and limitations of these methodologies. To conclude the review, we first offer some suggestions for interventions that could mitigate the effects of polarization, and second, point to directions for future research. We suggest expanding the national focus that has dominated the study of polarization, highlighting the benefits of cross-national comparisons with a global reach.

## Plain language summary

The study of political polarization has undergone significant changes, progressing from early emphasis on ideological divides toward a more complex understanding. This shift reflects the growing recognition that polarization is not merely disagreement about policy preferences, but something shaped by emotional, social, technological, and knowledge-related processes. In this review, we look at the various ways to approach the study of political polarization. A well-organized, comprehensive and interdisciplinary overview is surely necessary. We begin by elaborating different types of political polarization, moving from an early focus on ideological divides to more complex constructs. Then, we shift to an overview of key disciplinary concepts and theoretical approaches for examining political polarization. At the heart of this review, we elaborate the various methodologies and methods for studying polarization, including quantitative, computational/digital, experimental, and qualitative approaches. We also highlight the strengths and limitations of these methodologies. To conclude the review, we first offer some suggestions to mitigate the effects of polarization, and second, point to directions for future research. We suggest expanding the national focus that has dominated the study of polarization, highlighting the benefits of cross-national comparisons with a global reach.

In this review, we provide an elaboration of the various ways to approach the study of political polarization. This task may be daunting, but a well-organized, comprehensive and interdisciplinary overview is surely necessary. Part of the problem is that very good overviews have been published within specific disciplines ( Caluwaerts et al., 2023; Gidron et al., 2019; Kreiss & McGregor, 2024; Kubin & Sikorski, 2021; Lubej et al., 2025; Marino & others, 2024; Shah, 2025; Tucker et al., 2018; Wagner, 2024; Yair, 2020), but to our knowledge, no overarching summary exists.

We begin by elaborating different types of political polarization, moving from an early focus on ideological divides to multidimensional constructs. Then, we shift to an overview of key disciplinary concepts and theoretical approaches for examining political polarization. At the heart of this review, we elaborate the various methodologies and methods for studying polarization, including quantitative, computational/digital, experimental, and qualitative approaches. We also highlight the strengths and limitations of these methodologies. To conclude the review, we first offer some suggestions for interventions that could mitigate the effects of polarization, and second, point to directions for future research. We suggest expanding the national focus that has dominated the study of polarization, highlighting the benefits of cross-national comparisons with a global reach.

## Types of political polarization

Contemporary research on political polarization has moved beyond its early focus on ideological divides to embrace a more nuanced, interdisciplinary framework. The literature identifies a range of polarization types—ideological, affective, positional, and beyond—each capturing distinct but interrelated dimensions of division within political systems. These conceptualizations reflect the field’s evolution toward recognizing the emotional, identity-based, symbolic, and digital drivers of political conflict.

### Ideological polarization

The study of polarization traces its conceptual roots to the seminal work of Lipset, Rokkan, and their colleagues (1967), whose cleavage theory identified enduring lines of societal conflict structuring political competition in Europe. Their model of historical cleavages, class (workers versus owners), center–periphery, urban–rural, and Church–state, explain how ideological polarization became institutionalized through party systems and voter alignments. These foundational conflicts had established the structural and cultural bases of ideological divisions long before contemporary debates on affective or identity-based polarization. While later research has emphasized emotional and digital dynamics, the cleavage framework remains essential for understanding how durable social oppositions continue to underpin political polarization. Costa (2025) argues that polarization today is sustained not merely by ideological distance but by representational incentives: politicians strategically emphasize conflict to mirror their constituents’ partisan emotions, reinforcing cycles of affective division between elites and voters.

The early focus of studies on polarization refers to the widening ideological gap between different camps in a society - typically labeled liberals and conservatives. This form of polarization encompasses both *ideological sorting* and *issue polarization*, that is, the growing distance between groups in their positions on specific policy issues. The research agenda on ideological polarization begins with studying ideological polarization among political elites, especially in the U.S. Congress. For example, McCarty et al. (2006) and Theriault (2006) examine the growing ideological distance between political parties since the 1970s, largely due to the replacement of moderate legislators with ideologically extreme representatives. Extending this analysis to the electorate, Abramowitz, and Saunders (2008) have found increasing ideological divergence among voters, particularly on social and cultural issues. They utilize American National Election Studies (ANES) data to show that voters are increasingly polarized on issues related to social welfare and cultural values. Together, these lines of research connect the structural cleavages identified by Lipset et al. (1967) with the elite-level and mass-level ideological sorting processes that define contemporary polarization.

However, this approach has been challenged by Fiorina et al. (2008) by introducing the concept of *partisan sorting*—arguing that voters align their party affiliations with preexisting ideological beliefs, rather than becoming more ideologically extreme. Both sides of the debate use the same datasets, but they diverge in their interpretation of whether polarization reflects actual shifts in public opinion or elite-driven cues.

### Affective polarization

A conceptual shift occurs with the identification of *affective polarization,* the emotional and identity-based dimension of partisan hostility. Rather than focusing on issue-based disagreement, such a perspective highlights the growing emotional hostility among members of opposing partisan groups.

Abramowitz and Webster (2016) had been among the first to empirically capture this dimension using “feeling thermometer” scores from the ANES, showing a steady rise in negative feelings toward political out-groups. Iyengar et al. (2012) have expanded on this by integrating *social identity theory* ( Tajfel & Turner, 1979), arguing that partisanship has become a core aspect of individual identity. This framework explains partisan animosity not as ideological conflict per se, but as in-group favoritism and out-group derogation, often measured through stereotypes and social distance indicators (e.g., discomfort with inter-party marriages). Framing affective polarization as identity-based has prompted a wave of experimental studies, revealing that individuals often follow partisan cues regardless of the strength of the argument ( Druckman et al., 2013; Levendusky, 2013). Studies using behavioral games and Implicit Association Tests (e.g., Iyengar & Westwood, 2015) have demonstrated that polarization affects interpersonal behaviors, including trust and hiring preferences.

Comparative research has extended these insights beyond the United States. Reiljan (2020) provides one of the first systematic cross-national measures of affective polarization in European party systems, demonstrating that partisan animosity and social-identity dynamics are not unique to the U.S. context but also embedded in multiparty democracies. Wagner (2021) (2021) further refines this framework, showing how affective polarization varies with party fragmentation and coalition potential in European contexts. Together, these studies underscore that affective polarization operates differently when ideological diversity and coalition-building remain institutionalized features of politics.

Further, social and political psychology (SPP) has since become a dominant empirical framework, focusing on both *individual and contextual determinants* (e.g., need for closure, uncertainty avoidance) and *intra-individual mechanisms* (e.g., group centrism). Luttig (2018) posit that individuals with high epistemic needs are drawn to cohesive ideological groups, while Lees and Cikara (2020) have shown that inaccurate *intergroup meta-perceptions *, the belief that out-groups dislike one’s own group, can intensify polarization.

### Complementary and emerging forms of polarization

Ideological and affection polarization have been complemented with different and more nuanced definitions, enriching debates on the topic. Below, we offer a snapshot of various types of polarization.

***Partisan polarization*** refers to increasing alignment of political identities and behaviors along party lines; often overlapping with ideological and affective polarization. In this definition, competing political parties and their leaders trigger polarization in society ( Marino & others, 2024).

***Social polarization*** refers to the division of society into distinct groups. It involves the division of political attitudes, beliefs, and identities within a society, leading to a divergence in opinions, particularly along ideological, partisan, or cultural dimensions. This divergence can result in increased conflict among societal groups and reduced opportunities for compromise. Concepts like *affective economies* ( Ahmed, 2014) and *deep stories* ( Hochschild, 2016) explain how emotions circulate to reinforce “us vs. them” divisions.

***False polarization*** has been identified as a distinct type of political division. It is characterized by the perception of differences between groups that are exaggerated or falsely accentuated, rather than reflecting actual discrepancies in opinions or beliefs. It is also related to inaccurate and negatively biased judgments about opposing groups. Philosophical analyses suggest that such distortions result from *expressive responding*—individuals exaggerate disagreement to signal loyalty to their group, rather than to express genuine belief ( Hannon, 2021; Lees & Cikara, 2020).

This type of polarization is also related to *intergroup meta-judgment,* which refers to “what I think they think about us.” Studies have found that in competitive intergroup contexts, these group meta-perceptions are consistently inaccurate and negatively biased. This inaccuracy is linked to the (often false) perception that out-groups are deliberately obstructing collective action ( Lees & Cikara, 2020).

***Interpretative polarization*** is about the ways in which different social groups interpret and contextualize political events through divergent lenses. This form often reflects media influence and narrative framing and is particularly evident in polarized digital environments such as Facebook, Twitter and WhatsApp. Interpretative polarization implies that those frames used by one camp are deemed unfounded, inappropriate, or illegitimate by other camps. When interpretative polarization is strong, different groups conceptualize the same topic in vastly different terms, making meaningful conversation between groups almost impossible ( Kligler-Vilenchik et al., 2020).

***Interactional polarization*** describes the tendency, particularly pronounced in digital environments, for individuals to increasingly engage with like-minded others while avoiding contact with opposing viewpoints. This phenomenon contributes to the fragmentation of publics, limits opportunities for cross-cutting political dialogue, and undermines democratic negotiation and deliberation. It is often associated with the formation of “echo chambers,” in which users are algorithmically or behaviorally filtered into ideologically homogeneous networks that reinforce pre-existing beliefs and affective loyalties ( Bruno et al., 2022; Weber et al., 2022).

Ultimately, reduced cross-group interaction, whether through avoidance or defensive reactions, is correlated with heightened out-group hostility, increased stereotyping, and deeper ideological divides ( Rathje et al., 2023; Westwood et al., 2018). In their study of cross-platform engagement, Yarchi, Baden, and Kligler-Vilenchik (2021) show that social media ecosystems tend to foster both interactional and affective polarization, though the dynamics differ by platform architecture, user behavior, and the affordances for emotional expression. Their findings underscore that online polarization is not only a matter of what content people see, but also how they interact with others and emotionally respond to political differences in digital contexts.

***Horizontal polarization*** refers to the growing social and emotional distance between citizens of opposing political groups, marked by distrust, hostility, and moral disdain. It is measured through interpersonal evaluations of partisan supporters and affects social cohesion. Meanwhile, its opposite, ***vertical polarization*** captures attitudes toward political parties and elites, measured by feeling thermometers or like/dislike scores ( Bettarelli et al., 2022; Reiljan et al., 2024). Citizens often dislike opposing elites more than opposing voters. Studies show that these dimensions can coexist and reinforce one another ( Bettarelli et al., 2022; Harteveld, 2021).

***Nationalist polarization*** centers around nationalist narratives, often involving opposition to perceived outsiders and elites. This form of polarization is characterized by heightened animosity, distrust, and social distance between groups based on their differing views on national identity, who belongs to the nation, and related cultural issues. It is conceptually adjacent to populist discourse and is particularly salient in right-wing mobilization strategies ( Gidron et al., 2022; Woods et al., 2023).

***Performative polarization***, as conceptualized by Revers (2023), refers to the lived experience of division generated through political conflict. It is driven by *symbolic entrepreneurs*—political actors who seek to elicit disapproval from adversaries as a means of energizing and mobilizing their own supporters ( Ostiguy et al., 2020; Revers, 2023). This form of polarization is anti-universalist and escalatory, grounded in symbolic and affective performances rather than moral or ideological claims. Revers argues that it should be examined through everyday interactions and media discourse, employing interpretive and ethnographic methods capable of tracing the emotionally charged enactments that dramatize moral antagonisms.

Analytical philosophy examines ***polarization through deep and cross-disagreements **.* Deep disagreements involve conflicting normative frameworks, making resolution difficult and polarization persistent. Cross-disagreements arise when parties interpret the nature of a debate differently, leading to miscommunication and reinforcement of initial positions. Both hinder meaningful dialogue and are central to understanding the cognitive and emotional roots of polarization ( Ridder, 2021).

## Key disciplinary concepts and theoretical approaches

The academic understanding of political polarization has evolved from a narrow emphasis on ideological divergence to a complex, interdisciplinary exploration of emotional, identity-based, and digitally mediated dynamics. The literature now reflects a multifaceted view of polarization, with distinct but interrelated conceptual strands emerging from political science, cultural sociology, the sociology of emotions, philosophy, social psychology, and digital/visual communication theories.

### Political science

Political science has long engaged with polarization as a central tension within democratic governance, originally through the lens of ideological polarization. This early literature, particularly influential in the U.S. context, had highlighted increasing policy divergence among political elites, especially within the U.S. Congress, where moderates were increasingly replaced by ideologically extreme representatives ( Abramowitz & Saunders, 2008; McCarty et al., 2006). This elite-driven framework has been extended to the electorate, focusing on the ways in which citizens appeared to be adopting more polarized policy preferences. However, Fiorina et al. (2008) challenge this view, advancing the partisan sorting hypothesis, which contends that voters are not necessarily becoming more extreme but are increasingly aligning their partisan identities with preexisting ideological dispositions. This interpretation repositions polarization as an outcome of elite-led political cueing, rather than grassroots radicalization.

A paradigmatic shift in the field has occurred with the rise of affective polarization, which reorients attention from ideological disagreement to emotional hostility and social distance between partisan groups. Building on social identity theory ( Tajfel & Turner, 1979), Iyengar et al. (2012) conceptualize partisanship as a primary social identity, a framework through which individuals categorize themselves and others, often independent of specific issue positions. This identity-centric model has revealed how affective bonds with co-partisans and negative stereotyping of out-partisans can intensify, even in the absence of growing ideological distance. The implications are significant: polarization is no longer confined to matters of policy disagreement but extends into the social fabric of everyday life, influencing interpersonal trust, hiring decisions, and even the willingness to engage in intergroup relationships ( Druckman & Levendusky, 2019; Iyengar & Westwood, 2015).

This reconceptualization has catalyzed a diversification of theoretical frameworks and measurement strategies. In addition to social identity theory, scholars have incorporated motivated-reasoning theory, which explains how individuals selectively interpret information to protect their group identity and existing worldview. Others draw on intergroup emotions theory (IET) to account for the role of discrete, socially triggered emotions, such as anger, disgust, pride, and fear, which extend beyond binary like/dislike distinctions and help explain both attitudinal hardening and behavioral withdrawal ( Bakker & Lelkes, 2024). Meanwhile, a growing literature influenced by discursive institutionalism and cultural sociology views affective polarization as socially constructed and performed, emphasizing the role of elite discourse, partisan media, and symbolic boundaries in reproducing “us versus them” imaginaries ( Erdoğan & Uyan Semerci, 2025; Kim et al., 2024; Trigiani & Boler, 2021).

This theoretical pluralism has produced a rich, but fragmented, array of measurement approaches. Traditional tools, such as feeling thermometers, remain widely used in surveys like ANES, but are increasingly complemented by social distance scales, trust metrics, survey experiments, and computational text analysis using sentiment and toxicity classifiers. In multiparty systems, composite indices, such as Reiljan’s (2020) Index of Affective Polarization, integrate multiple dimensions, including leader aversion, symbolic antagonism, and moral superiority. At the same time, qualitative methodologies, including discourse analysis, digital ethnography, and in-depth interviews, offer insights into how individuals narrate, experience, and emotionally invest in partisan divides. While this methodological diversification has enhanced empirical reach, it also presents challenges of conceptual coherence, cross-study comparability, and context-sensitive validity issues that future research must address to consolidate affective polarization as a robust and generalizable construct within political science.

### Social psychology

In the field of social psychology, polarization is understood not merely as a divergence on policy issues, but as an umbrella concept encompassing affective distance and growing social differentiation between groups. Grounded heavily in Social Identity Theory ( Tajfel & Turner, 1979; Iyengar et al., 2019), this perspective posits that group membership is a primary source of self-esteem, motivating individuals to favour their in-group and derogate the out-group. Crucially, social psychology distinguishes between actual polarization (real discrepancies in attitudes) and perceived polarization (falsely accentuated or subjectively imagined differences), identifying the latter as a potent driver of intergroup hostility.

The social psychological perspective is unique due to its emphasis on quantitative and indirect measurement techniques. As highlighted in recent field reviews, psychologists seldom assess polarization by directly examining differences in policy preferences ( Martherus et al., 2021). Rather, they define it through indirect psychological concepts, particularly affective polarization (emotional separation) and group meta-perceptions (assumptions about the opposing side’s thoughts). This methodological focus shifts the diagnosis of the problem: polarization is treated less as a disagreement over facts and more as a distortion in social cognition, where the ‘reality’ of the conflict matters less than the psychological representation of the opponent.

Research on the determinants of polarization has evolved from simple personality traits to complex cognitive-motivational mechanisms. As outlined by Jost et al. (2022), polarization is driven by three distinct classes of motives: ego-justifying (defending one’s positive self-image), group-justifying (advancing in-group interests), and system-justifying (defending the status quo). While earlier hypotheses like the “group centrism” model ( Luttig, 2018) had suggested that a high need for closure drives individuals toward homogeneous groups, recent work points to dynamism. For instance, Garrett and Bankert (2020) demonstrate that moral conviction acts as a distinct accelerant; when political preferences are moralized, they become non-negotiable, significantly heightening affective polarization independent of partisan identity strength. Similarly, Morisi et al. (2020) find that relational motives, specifically the desire to maintain in-group homogeneity fuel confirmation bias, particularly among conservatives.

However, a growing body of work challenges the “symmetrical” view that liberals and conservatives are equally driven by tribal instincts. The theory of ideological asymmetry ( Jost, 2017) suggests that the psychological drivers of polarization differ across the ideological spectrum. While conservatives are often motivated by an epistemic need for certainty and system justification (defending the status quo/order), liberals are more often motivated by a tolerance for ambiguity and a drive for egalitarian social change. This suggests that polarization is not a uniform psychological reflex; rather, “system-threatening” information triggers defensive polarization more intensely in those with high needs for order, whereas “inequality-justifying” information triggers it in those with high needs for equality.

Beyond individual traits, research emphasizes how contextual triggers and “collective psychology” escalate polarization. Levin and Weber (2023) argue that polarization spikes when collectives switch from calculation-based decision modes (rational, fact-oriented) to affect-based modes (driven by identity and emotion). Digital environments are particularly prone to triggering this shift. Brady et al. (2017) find that the use of moral-emotional language, like “betrayal” or “shame” acts as a contagion mechanism. This language serves less as a tool for persuasion and more as a signal of group loyalty, reinforcing in-group cohesion while amplifying out-group antagonism. Consequently, the communicative environment itself can lock groups into feedback loops of hostility, regardless of the actual ideological distance between them.

Amidst these dynamics, social psychologists emphasize the need to distinguish *who* is the target of this hostility. Kingzette (2021) argue that treating the out-group as a monolith obscures crucial differences between feelings toward political elites versus ordinary voters. Their research suggests that while hostility toward opposing politicians may be a rational response to genuine policy disagreements, hostility toward ordinary voters represents a more dangerous “spillover” effect. This distinction is vital because democracy requires holding elites accountable (which may require negative affect), but it is threatened when that animosity is transferred to everyday social interactions, leading to the severance of ties between citizens.

Zooming in on the intra-individual level, studies identify the specific mechanisms that transform ordinary disagreement into ‘toxic’ polarization. Moore-Berg et al. (2020) identify the “prime suspects” of this toxicity: dehumanization and meta-perceptions. Dehumanization occurs when partisans view opponents as lacking essential human traits (such as rationality or moral sensibility), which erodes the inhibition against harming them. Closely linked to this are meta-perception or “what I think they think about us.” Lees and Cikara (2020) find that in competitive contexts, these meta-perceptions are consistently inaccurate; partisans vastly overestimate the level of hatred the out-group holds toward them. This “phantom polarization” creates a self-fulfilling prophecy, where imagined hostility from the other side justifies actual hostility from one’s own side.

Despite the clarity of these psychological mechanisms, recent large-scale experiments have cast doubt on the assumption that fixing them will automatically restore democratic health. Voelkel et al. (2023) and Broockman et al. (2023) demonstrated that while interventions can successfully reduce affective polarization (making partisans “feel” warmer toward each other), these shifts rarely translate into changed political behaviour or reduced support for undemocratic candidates. This “disconnect” suggests that affective polarization may be a symptom rather than the primary cause of democratic backsliding. It implies that psychological animosity, while toxic, acts independently of the deeper illiberal attitudes that threaten democratic institutions.

Finally, recent contributions from critical social psychology have begun to question the normative assumption that polarization is inherently a dysfunction. Scholars like Balinhas (2023) argue that the mainstream focus on reducing polarization can inadvertently serve to depoliticize legitimate social grievances. By framing strong disagreement as a psychological bias (e.g., “tribalism” or “affective hate”) rather than a substantive dispute over power and justice, researchers risk masking power asymmetries. From this critical perspective, what appears to be “toxic polarization” may sometimes be the necessary psychological mobilization of marginalized groups challenging a dominant status quo. Complementing this view, Binder et al. (2025) propose viewing polarization through the lens of cultural sociology, describing it not just as an emotional bias but as a “social drama” or a struggle over meaning. Thus, what psychologists diagnose as “toxicity” may alternatively be understood as the necessary friction of a society actively renegotiating its collective identity and moral values.

### Cultural sociology

Cultural sociological frameworks approach polarization by focusing on the meanings, values, identities, and emotional dynamics that shape political divides within a society. This perspective often contrasts with more traditional views that might emphasize economic cleavages or purely ideological differences, instead delving into the “inner feeling structures of polarized issues.”

First, Laclau’s (2005a, 2005b) conceptualization of populism involves political logic rather than a fixed ideology; it constructs antagonisms between “the people” and “the elites,” often through symbolic appeals to empty signifiers like “freedom” or “justice.” Rather than having a fixed, singular meaning, these signifiers can be filled with various meanings depending on the context and the specific grievances being articulated. Polarization, in this view, arises from the mutual constitution of antagonist discourses, which introduces a cleavage within “the people” itself, shifting political landscapes through symbolic and discursive means ( Urbinati, 2019).

Second, contrary to other approaches stigmatizing polarization as a societal anomaly, Jeffrey Alexander (2019) posits that polarization is a “normal” phenomenon within civil spheres of democracies; however, it becomes a fundamental danger when the cultural premises and structural foundations of civil solidarity are challenged. “Frontlash” refers to “forward-thinking” movements that spearhead progress and introduce reforms. “Backlash” movements are reactions of “cultural, social, and political un-doing” that aim to “unwind cosmopolitan widening and civil incorporation.” Alexander argues that backlash is inevitable when frontlash movements destabilize established interests, but the critical question for democracy is whether the civil sphere can survive these counter-movements. This framework is rooted in the “Strong Program” in cultural sociology, which aims to reconstruct meaning making and reveal underlying “culture structures,” asserting the “relative analytical autonomy” of culture as an independent causal variable ( Alexander & Smith, 2003). Structural hermeneutics, the method inspired by the Strong Program, emphasizes the reconstruction of meaning-making processes and underlying cultural codes as causal forces in polarization. This lens helps explain how even opposing narratives (e.g., pro- vs. anti-science) often draw from shared symbolic binaries.

Another way that a cultural sociological approach examines polarization is through the lens of the “authority of science” and how it is perceived by the public. Houtman, Aupers, and Laermans (2021) suggest moving beyond a “good” vs. “bad” perspective on trust in science to reveal the cultural mechanisms that lead people to accept or deny its authority. This includes viewing rejections of science as rejections of “scientism,” a secular religion. Similarly, Harambam and Aupers (2021) demonstrate how cultural sociology reveals the ways in which conspiracy theorists engage with science, showing that they are not necessarily “anti-science” but may relate to science in “complex, nuanced, and ambivalent” ways. Both mainstream and alternative narratives, including “performative conspiracy” theories related to issues such as COVID-19, often draw upon the same underlying binaries (e.g., science vs. blind faith, truth vs. deception, evidence vs. speculation), which can be analytically identified as precise points of polarization ( Jaworsky, 2023).

Finally, Matthias Revers (2023) conceptualizes performative polarization as the lived and observed experience of political dividedness, rooted in the symbolic and emotional dynamics of public conflict. Unlike moral or universalist framings, performative polarization is distinctly anti-universalist, focusing not on shared values but on dramatized confrontation. It is driven by symbolic entrepreneurs, actors who intentionally provoke disapproval from opposing audiences to generate emotional energy, which in turn consolidates and empowers their own base. This process operates through a logic of escalation, whereby increasingly dramatic acts and symbolic associations deepen existing divisions by linking new issues to entrenched grievances. Revers’ approach aligns with cultural sociology’s emphasis on meaning-making in public life.

### The sociology of emotions

The sociology of emotions offers a compelling framework for understanding political polarization as a process deeply rooted in emotional meaning-making, cultural scripts, and social structure. Rather than treating emotions as irrational byproducts of ideological or economic conflict, this perspective recognizes them as constitutive forces socially regulated, contextually embedded, and central to identity formation. Drawing on Arlie Hochschild’s foundational work on *“feeling rules”* and *“deep stories”* ( Hochschild, 1983, 2016), scholars examine how individuals narrate their place in the world through emotionally charged accounts of group belonging, injustice, and moral worth. Emotions are conceptualized not as binary opposites to reason, but as forms of situated knowledge, produced within and through power relations, cultural expectations, and social hierarchies ( Ahmed, 2014; Salmela & von Scheve, 2017).

This approach is particularly attuned to how emotions such as anger, fear, disgust, pride, and resentment are mobilized within polarized discourses and institutional settings. Through concepts like “affective economies” ( Ahmed, 2014), emotions are understood as circulating between bodies, media, and institutions, attaching value to identities, symbols, and ideologies, and thereby sustaining the emotional architecture of “us” versus “them” divisions. These emotional attachments are not merely expressive; they are performative and political, shaping group cohesion, boundary formation, and moral positioning. Unlike standard measures of affective polarization (e.g., “feeling thermometers”), which reduce emotion to quantifiable intergroup animosity, the sociology of emotions interrogates the qualitative texture of emotional life how affect is experienced, displayed, legitimated, or silenced across political, cultural, and media contexts ( Bericat, 2015; Jaworsky, 2023).

Crucially, this framework also exposes how epistemic hierarchies are maintained through the dichotomy between emotion and reason. As Durnová (2015) argues, in polarized public discourse, emotional expression, especially in the form of public outrage is frequently delegitimized as “irrational,” while scientific expertise is upheld as the sole locus of rational authority. This framing not only excludes dissenting voices but also “weaponizes truth”, deepening division by pathologizing emotional disagreement. In a “post-truth” era, where factual authority and emotional resonance compete for legitimacy, recognizing emotions as valid epistemic claims may offer an alternative pathway to democratic inclusion and depolarization. By illuminating how emotional dynamics are culturally and structurally produced, the sociology of emotions provides a rich, interpretive complement to psychological and computational models, emphasizing that polarization is not only a matter of cognition or ideology, but also of feeling, belonging, and recognition.

### Philosophical perspectives

Philosophical perspectives on polarization primarily delve into the norms of behavior of individuals or groups in relation to the beliefs they hold and the attitudes with which they confront those beliefs. This approach aims to extrapolate these norms and apply them across different discussion contexts. Unlike sociological or political science perspectives that study political parties or public opinion, philosophy analyzes the fundamental norms governing beliefs and attitudes. Philosophical analysis is less concerned with the political content of beliefs and opinions and more with classifying these positions in epistemological terms. Its normative assessment is based on scenarios of individual and collective knowledge formation rather than democratic normativity or ideal public space functioning.

Philosophers are fundamentally concerned with evaluating the epistemic validity and normative justification of beliefs, particularly in contexts of political and moral disagreement. This approach distinguishes philosophical inquiry from other disciplines such as political science, sociology, or psychology, which typically focus on how beliefs are formed, circulated, or emotionally experienced, rather than whether they are rationally warranted. In addressing polarization, philosophers investigate whether individuals in opposing camps have good reasons for their views, or whether those views result from epistemic failures such as motivated reasoning, closed-mindedness, or the misapplication of moral principles. This concern with the standards of rational belief leads philosophical approaches to frequently draw on empirical research from social and cognitive psychology, particularly experimental studies that isolate cognitive biases, situational factors, and heuristic errors in judgment formation ( Kelly, 2008; Ridder, 2021).

However, philosophers are not merely consumers of psychological data, they use these data to assess the conditions under which beliefs can be considered justified or unjustified, and what this means for democratic discourse. For example, they interrogate whether polarized beliefs can be rationally sustained in the face of contrary evidence, or whether deep ideological divides reflect more intractable “epistemic impasses” ( Almagro, 2023). These are not simply communication failures, but structural clashes in normative frameworks, where even agreement on facts may not lead to consensus due to divergent conceptions of evidence, authority, or moral obligation. In this sense, philosophical contributions help clarify what constitutes reasonable disagreement, when belief polarization crosses into epistemic irresponsibility, and how pluralistic societies might rebuild epistemic trust without succumbing to relativism or coercion.

Philosophy provides a precise re-conceptualization of different types of polarization, enabling a more accurate diagnosis of their underlying causes. Osorio and Villanueva (2019) analyze how parties’ positions may not always refer to the same object of dispute, and how evaluative language expresses not just beliefs but also underlying attitudes. Almagro (2023) characterizes radicalism by an increased reliance on one’s own group’s beliefs, rather than merely adopting extreme positions. This positioning aligns with the concept of “immune beliefs,” in which in-group beliefs are protected against external reasons, and with attitudes like pathological confidence or epistemic arrogance. Identifying these emotions and epistemic vices can inform prevention and neutralization strategies.

Philosophical analysis also challenges the measurement of polarization through survey items. It raises the question of whether political statements in polarized contexts reflect genuine beliefs (a cognitivist reading) or merely the expression of desires, political loyalty, or preference (a non-cognitivist reading). As suggested by Hannon (2021), political polarization is not a true disagreement of beliefs but a form of “expressive responding,” through which people exaggerate disagreement to reinforce group loyalty rather than reflecting genuine differences on facts or policies. Meanwhile Almagro (2023) questions whether self-assigned beliefs (obtained via surveys or interviews) accurately reflect an individual’s state of mind. Discursive expressions in polarized contexts may not reflect genuine beliefs but rather non-cognitive attitudes designed to reinforce group cohesion. Instead, Almagro proposes a framework based on political commitment and Lynch’s “political meaning,” ( Lynch, 2018) in which self-reports reveal an individual’s degree of commitment to core group beliefs. This philosophical approach offers a “corrective methodology” to empirical studies by focusing on the authenticity of beliefs and rationality.

Political polarization often arises from two distinct types of disagreements ( Kelly, 2008; Ridder, 2021). “Deep disagreements” extend beyond mere factual disputes, centering instead on fundamental differences in the normative frameworks used to resolve issues. When opposing parties cannot agree on the very principles by which a resolution should be reached, polarization is likely to persist. Another significant contributor is “cross disagreement,” in which the nature of the disagreement itself is interpreted differently by the involved parties. For example, one side might perceive an issue as purely factual, while the other understands it through a normative lens. This disparity creates an “illusion of dialogue,” where arguments fail to be understood or to influence the opposing side. As participants entrench their original positions, this type of disagreement intensifies polarization. Osorio and Villanueva (2019) have explored how cross-disagreements contribute to this phenomenon, often observing that political allegiance becomes an expression of group membership rather than a cognitive description.

### Digital and visual communication theories

Digital and visual communication theories approach polarization by focusing on how modern media environments, particularly social media platforms, shape and amplify political divisions through specific content formats and algorithmic processes. The rise of social media platforms like Twitter (X), Facebook, and TikTok has profoundly impacted how political polarization manifests in the digital age. These platforms facilitate the formation of distinct online communities and echo chambers, in which users are primarily exposed to information that aligns with their existing viewpoints. This solidarity reinforces partisan identities and shields users from opposing perspectives, thereby contributing to greater polarization. Research has shown ideological polarization between Twitter users and the existence of echo chambers through analysis of millions of tweets ( Barberá et al., 2015). The prevalence of tailored content on social media exacerbates the disconnect between individuals from opposing political viewpoints, further entrenching societal divides.

Research in this discipline has also revealed that platform-specific algorithms play a significant role in amplifying polarization. Algorithmic filters contribute to the creation of echo chambers that reinforce existing beliefs and preferences. Algorithm-driven content exacerbates affective polarization by amplifying emotionally charged content. There is an ongoing discussion about whether technologies like AI and algorithms represent primary drivers of polarization or if they hold potential for mitigation ( Kim et al., 2024; Yarchi et al., 2021).

Automated activity, such as the use of bots, also represents a critical area of focus, not least because it disseminates misinformation and increases polarization online. Bots are estimated to account for a significant portion of online content (e.g., one in five tweets, up to 15 percent of active Twitter/X users) and primarily amplify low-credibility online material ( Caldarelli et al., 2020; Linvill & Warren, 2020). Experiments, including simulations, have demonstrated that even a single bot can shift average population opinion towards more extreme positions, increasing polarization without direct human-bot interaction. Bot activity often escalates close to major events like elections, amplifying the voices of a minority of users and driving online narratives, especially by retweeting non-credible information from genuine users to give it credibility. Bots contribute to the narrative by using frames that appeal to emotions (e.g., sympathy or humor) ( Bruno et al., 2022; Daume et al., 2023; Weber et al., 2022).

Large language models (LLMs), such as those developed by OpenAI, are increasingly deployed in polarization research to analyze large-scale digital communication, detect patterns of affective and ideological division, and simulate possible trajectories of partisan sorting. These AI systems are capable of extracting moral-emotional language, identifying affective markers, and categorizing political sentiment across vast datasets from platforms such as Twitter/X, YouTube, and TikTok ( Martin-Gutierrez et al., 2024; Peng & Lu, 2023). In addition to serving as analytical tools, LLMs are also being tested as synthetic agents, such as GPT-powered bots that engage in dialogue to de-escalate polarization and promote perspective-taking ( Govers et al., 2024).

However, AI-generated content particularly when used to amplify or fabricate misinformation, poses significant risks to the integrity of political discourse. Research suggests that the emotional intensity of user reactions to AI-generated or algorithmically curated content plays a decisive role in determining its polarizing impact ( Caldarelli et al., 2020; Pescetelli et al., 2022). This finding aligns with broader research in social and political psychology showing that algorithm-driven amplification mechanisms tend to prioritize emotionally provocative content, such as anger, fear, or moral condemnation, enhancing both visibility and engagement, and thereby reinforcing affective divides ( Brady et al., 2017; Kim et al., 2024; Yarchi et al., 2021).

The multimodal nature of digital platforms, which integrate images, sound, music, and text further intensifies the emotional salience of political messaging. Studies have shown that the combination of visual memes, dramatic music, and charged textual commentary contributes to the viral spread of polarized narratives and strengthens in-group emotional bonding ( Hunter, 2023; Łabuz & Nehring, 2024; Rashid et al., 2024). These dynamics underscore the importance of understanding emotional expression and performance in digital spaces—not merely as reflections of polarization but as drivers of it, embedded within both algorithmic systems and human interpretive behavior.

Recent research on visual political polarization underscores the growing significance of imagery in constructing political meaning and reinforcing ideological divides. In contemporary digital environments, visual communication, including photos, graphics, memes, and video clips, has become a dominant mode through which political narratives are framed and affectively charged. This trend is especially salient on multimodal platforms like TikTok, in which the integration of visuals, music, and text amplifies the emotional resonance of political messages, enhancing their persuasiveness and, in many cases, their polarizing effects ( Kim et al., 2024; Łabuz & Nehring, 2024; Moffitt, 2022). Rather than simply reflecting pre-existing views, these platforms actively shape how political emotions, such as fear, pride, or resentment, are performed and circulated.

A central focus in this literature is the visual strategy and image management employed by political actors. Politicians increasingly curate their public persona through emotionally resonant and culturally symbolic imagery particularly on visual-first platforms like Instagram, to influence voter perception, signal group identity, and mobilize partisan sentiment. Research by Bast (2021) and De-Lima-Santos et al. (2023) demonstrates how political figures utilize themes such as national identity, family values, and authenticity to craft highly stylized visual narratives that resonate with their target audiences. This practice, known as visual political image management, has become a critical mechanism for constructing affective proximity with supporters while distinguishing the political “self” from ideological opponents.

Importantly, the design choices embedded in visual framing, including composition, color schemes, symbols, and contextual cues can either intensify polarization or foster common ground, depending on how they position viewers in relation to the content. As Kubin and von Sikorski (2024) argue, visual cues can serve not only as triggers of emotional outrage but also as tools for depolarizing interventions, particularly when embedded within strategies like content warnings or inclusive framing. However, the reception of such visual cues is mediated by individual trust in media sources. Research shows that audiences with low institutional trust are more likely to dismiss or critically interpret visual content, even when it aligns with their ideological position, further complicating the role of imagery in political persuasion ( Yarchi et al., 2021).

## Methodologies and methods

In this section, we categorize and sort the myriad of methodologies and methods utilized to study polarization. Of course, some of the ways in which we have sorted them are necessarily arbitrary; for example, a method may be both quantitative and experimental. However, we believe that simplifying into several broad types of methodology helps to organize an increasingly messy literature. Accordingly, we speak about quantitative, experimental, digital and computational, and qualitative approaches. For each, we provide a summary of the strengths and limitations of employing a particular approach.

### Quantitative methodologies and methods

Quantitative research has played a foundational role in measuring both ideological and affective polarization. Political scientists had first identified increasing ideological distance among U.S. legislators using roll-call vote data ( McCarty et al., 2006; Theriault, 2006). This approach has been extended to the mass public through survey-based studies using American National Election Studies (ANES) data, which reveals similar partisan sorting and ideological polarization among voters ( Abramowitz & Saunders, 2008). Social and political psychologists have advanced the study of polarization by introducing the concept of affective polarization, which refers to the growing emotional hostility between political groups, independent of ideological distance. To empirically measure this phenomenon, researchers have developed a set of tools including “feeling thermometer” scores, social distance metrics, and out-party stereotypes ( Iyengar et al., 2012; Iyengar & Westwood, 2015). Feeling thermometer scores assess respondents’ emotional warmth or hostility toward political groups by asking them to rate parties or partisans on a scale typically ranging from 0 (coldest feelings) to 100 (warmest feelings). A large gap between ratings for in-party and out-party signals high affective polarization. Social distance metrics capture respondents’ willingness to engage in various forms of interpersonal relations with members of the opposing political group such as marrying into the group, working with them, or living nearby. Greater reluctance to interact with out-party members is indicative of increased affective distance. Out-party stereotyping involves evaluating the extent to which individuals hold negative and homogenized beliefs about members of the opposing political party (e.g., seeing them as unintelligent, immoral, or unpatriotic). These stereotypes are often resistant to correction and reflect deep-seated partisan animosity.

***Cross-national methods***

Cross-national research has increasingly demonstrated that affective polarization is not confined to two-party systems like that of the United States, but also significantly shapes political dynamics in multiparty democracies. This insight has been facilitated by the development of comparative datasets, most notably the Comparative Study of Electoral Systems (CSES), which provides harmonized public opinion and electoral data across a wide range of countries. Building on this foundation, Reiljan (2020) has introduced the Index of Affective Polarization (API), a cross-national metric designed to quantify the emotional distance between partisans in multiparty contexts. The API measures the average affective evaluations that supporters of each party express toward all other parties, weighted by vote shares, thereby capturing not only bilateral animosity (as in two-party systems) but the broader emotional topology of the party system. Reiljan’s findings reveal that affective polarization is a pervasive phenomenon across democracies, manifesting through generalized negative feelings toward out-parties and not simply as ideological distance. This challenges the U.S.-centric assumption that partisan hostility is a product of binary opposition and instead suggests that affective partisanship is structurally embedded even in pluralistic political environments, often influenced by elite rhetoric, media systems, and cultural context. By enabling comparative analyses, such indices underscore the global relevance of affective polarization and provide tools for exploring how its intensity and drivers vary across institutional configurations, party systems, and sociopolitical histories.

***Political science methods***

In political science, quantitative approaches to polarization often focus on the behavior of political elites, patterns of partisan alignment among the electorate (known as partisan sorting), and the increasing divergence in policy stances between ideological groups. To analyze these phenomena, researchers rely on various methods of operationalization, including policy preference scales, vote share analysis, and party manifesto data. Policy preference scales typically derive from survey instruments that ask respondents or, in elite studies, legislators, to position themselves on a range of salient political issues (e.g., immigration, taxation, climate policy). These responses are then aggregated to estimate ideological distance between parties or individuals. Prominent examples include the use of Likert-type scales or comparative voter surveys like the Chapel Hill Expert Survey or CSES. Vote shares refer to the proportion of electoral support received by parties or candidates, often used to track shifts in public alignment or electoral volatility. When mapped over time, changes in vote shares can reveal patterns of increasing support for ideologically extreme parties, or the erosion of centrist consensus, both of which are indicators of polarization. Party manifestos are systematically analyzed texts in which political parties outline their policy priorities and ideological commitments. Quantitative content analysis tools, such as the Comparative Manifesto Project (CMP) assign scores to policy positions and track issue emphases over time. These scores are then used to estimate ideological distances between parties and assess whether systems are becoming more polarized on key dimensions (e.g., left–right, economic vs. cultural issues). These operational tools enable political scientists to quantify ideological and positional polarization, particularly at the elite and party-system levels, thereby allowing them to examine how institutional and electoral structures shape the evolution of political conflict over time and space.

***Psychology methods***

In contrast to political science’s focus on systemic and elite-level indicators, psychology, particularly social and political psychology investigates polarization through the lens of intra-individual mechanisms, cognitive styles, and emotional dispositions. These approaches seek to uncover the psychological underpinnings of how individuals process political information, form group attachments, and develop hostility toward ideological opponents. A central methodological tool in this field is the use of psychometrically validated scales, which allow researchers to reliably measure stable psychological traits and state-based attitudes that contribute to polarized thinking and behavior. Key constructs often measured include:

• ***Need for Closure (NFC):*** A cognitive-motivational variable reflecting an individual’s desire for definitive answers and discomfort with ambiguity. High NFC has been shown to correlate with stronger in-group identification and greater susceptibility to black-and-white thinking, which in turn fosters affective polarization ( Luttig, 2018; Webster & Kruglanski, 1994).
• Authoritarianism, often measured through the ***Right-Wing Authoritarianism (RWA)*** scale, captures preferences for social conformity, hierarchical order, and submission to perceived legitimate authorities. High RWA scores correlate with heightened threat sensitivity, out-group derogation, and support for punitive policies, all of which contribute to ideological entrenchment and resistance to pluralism ( Altemeyer, 1996; Duckitt & Sibley, 2010).
• Similarly, ***Social Dominance Orientation (SDO)*** reflects a preference for intergroup hierarchy and opposition to egalitarianism. Individuals scoring high in SDO tend to endorse narratives that justify in-group superiority and out-group exclusion, thereby exacerbating polarization along racial, ethnic, or nationalist lines ( Federico & Sidanius, 2002; Pratto et al., 2006).
• ***Affective bias and partisan stereotyping**:* Experimental and survey-based tools are used to assess the extent of partisan affect, such as dislike of opposing-party members, willingness to socially distance from them, or endorsement of negative stereotypes. These measures are often linked to social identity theory, which views partisanship as a core component of self-concept, thus intensifying emotional responses to political stimuli ( Iyengar et al., 2012; Iyengar & Westwood, 2015).
• ***Moral foundations and emotional dispositions*:** Other psychological instruments evaluate how individuals’ moral intuitions (e.g., care/harm, fairness/loyalty) and affective tendencies (e.g., trait anger or disgust sensitivity) shape polarized responses to political content. For instance, moral conviction has been found to increase resistance to compromise and justify intolerance toward opposing views ( Clifford, 2020; Skitka et al., 2005).
• ***Stereotype Content Model (SCM):*** The SCM posits that group stereotypes are organized along two universal dimensions—warmth and competence—which predict distinct emotional and behavioral responses toward social groups. High-warmth, high-competence groups elicit admiration, whereas low-warmth, low-competence groups evoke contempt ( Cuddy et al., 2008; Fiske et al., 2002). This framework explains ambivalent prejudices and intergroup biases across cultures.

***Sociology methods***

Quantitative sociological approaches to polarization foreground the role of social structure, group identity, and cultural conflict in shaping political divisions. Rather than focusing solely on elite behavior or individual psychological traits, sociologists examine how macro-level inequalities and institutional arrangements generate and reinforce cleavages that become politically salient. These approaches emphasize that polarization is not just a matter of ideological divergence but is deeply embedded in the dynamics of social stratification, collective identity formation, and symbolic boundary-making.

To empirically investigate these dynamics, sociologists studying polarization often rely on quantitative indicators such as education level, class background, and religious affiliation to explore how political attitudes intersect with broader social cleavages. Education level is frequently used as a proxy for cultural capital and value orientation. For instance, Marino et al. (2024) highlight how education correlates with differentiated political preferences, contributing to a growing divide in political behavior between higher- and lower-educated citizens, especially in Western democracies. This pattern underpins what some describe as an education-based cultural cleavage, where cosmopolitan and liberal orientations contrast with more traditional or authoritarian ones. Class background and economic insecurity are captured through measures such as income, occupational status, and perceptions of precarity. These indicators are critical to understanding material grievances that often fuel resentment-driven or populist mobilization. The systematic literature analysis by Marino et al. (2024) further identifies economic status as a structural factor influencing ideological divides and voter alignment. Religious affiliation and religiosity serve as enduring cultural markers and often intersect with partisan identification, particularly on moral and social issues. Kahl et al. (2019) discusses how religion functions not just as a belief system but as an emotional and identity-based anchor in polarized debates, reinforcing ideological positions through shared norms and affective bonds. Moreover, recent work has highlighted the role of intersectionality, the interaction of race, gender, class, and other identities in shaping political polarization, showing that these dimensions do not operate in isolation but often reinforce one another within specific socio-political contexts ( Sahu & Sahu, 2024).

The study of political polarization has evolved, with quantitative surveys increasingly exploring its correlates such as authoritarian predispositions, epistemic closure, and belief in misinformation ( Garrett & Bankert, 2020). Researchers have utilized panel data to track individual-level polarization over time, frequently connecting it to emotional, cognitive, and identity-based factors ( Yarchi et al., 2021). Concurrently, political sociology has applied quantitative methods to investigate social cleavages, disparities between elites and the public, and how institutional mechanisms structure conflict.

***Strengths and limitations of quantitative methods***

Quantitative methods offer significant strengths in the study of political polarization, particularly in their ability to identify macro-level trends and enable cross-national comparisons. Large-scale surveys and datasets, such as the Comparative Study of Electoral Systems (CSES) allow researchers to systematically track ideological and affective divides over time and across diverse contexts ( Reiljan, 2020). Techniques such as vote share analysis, policy preference scaling, and manifesto coding enable scholars to measure elite polarization, partisan sorting, and issue alignment with precision ( Marino et al., 2024). In social and political psychology, experimental designs and psychometrically validated scales (e.g., for affective bias, need for closure, or authoritarianism) help identify intra-individual mechanisms that contribute to polarization, providing causal insights through statistical modeling ( Lees & Cikara, 2020).

However, these methods also face several important limitations. Quantitative approaches often rely on indirect measurements, such as like-dislike thermometers or social distance metrics, which may fail to capture the deep emotional, cultural, and symbolic dimensions of polarization. As scholars in the sociology of emotions point out, polarization is not only ideological but affective and identity-driven, often shaped by culturally embedded “feeling structures” that quantitative tools struggle to represent. Moreover, experimental studies, despite their internal validity, frequently suffer from low ecological validity and rely on convenient rather than representative samples, raising concerns about generalizability ( Lees & Cikara, 2020). Self-reported beliefs may also be distorted by expressive responding or group-signaling, leading to misinterpretations of the authenticity of respondents’ political attitudes ( Almagro, 2023; Hannon, 2021).

Additionally, quantitative research tends to be overrepresented in Western contexts, limiting its global applicability. Most survey instruments and conceptual frameworks have been developed in the United States or Europe, potentially missing the specific dynamics of polarization in non-Western societies ( Marino et al., 2024). Furthermore, philosophical critiques emphasize that many political disagreements are rooted in deep moral or epistemological differences, which resist simplification into numeric variables or standardized survey items ( Kelly, 2008; Ridder, 2021). While quantitative methods remain indispensable for large-scale and comparative research, they are most effective when combined with qualitative, discursive, or interpretive approaches that can illuminate the emotional, symbolic, and contextual layers of polarized political life.

### Experimental methods

Experimental methods, prevalent in psychology and behavioral economics, are crucial for pinpointing the causal mechanisms of political polarization. These methods involve manipulating specific variables within controlled environments, often employing randomized controlled trials (RCTs), laboratory experiments, and online platforms such as survey panels or social media simulations to gauge individual responses to political stimuli ( Druckman et al., 2013; Levendusky, 2013).

***Identity priming and messaging***

A key technique is identity priming, which subtly reminds participants of a superordinate identity (e.g., national identity or shared humanity) before they engage with partisan content. For example, Levendusky (2018) shows that priming individuals to identify as “Americans first” reduced affective polarization by diminishing partisan salience. Another strategy, value affirmation, prompts participants to reflect on core personal values, such as fairness or compassion. This approach has proven effective in reducing defensiveness when individuals encounter challenging viewpoints. Both identity priming and value affirmation aim to buffer individuals’ identities, making them more receptive to opposing perspectives.

Exposure to counter-attitudinal messages is another experimental approach, presenting participants with arguments or narratives from the opposing political side. These messages are meticulously crafted to be empathetic, credible, and relatable, thereby increasing the likelihood of reducing out-group animosity. Furthermore, misperception correction interventions address inaccurate beliefs about opposing partisans by providing accurate data regarding their beliefs, values, or behaviors. This can demonstrate, for instance, that political opponents are less extreme or hostile than imagined ( Lees & Cikara, 2020).

These experimental techniques are frequently integrated into pre-post designs, between-subjects comparisons, or longitudinal follow-ups to evaluate both the immediate and lasting effects of interventions. When conducted as online experiments via platforms like Prolific or Qualtrics, researchers can access diverse and scalable samples, though this may sometimes compromise ecological validity. Overall, these methods enable scholars to precisely identify the mechanisms driving polarization and to test targeted depolarization strategies under controlled and replicable conditions.

***Behavioral economics***

Behavioral economics contributes to polarization research by employing experimental paradigms such as the Dictator Game and Trust Game, which are used to study how partisan identity influences behavior even in non-political or apolitical contexts. In the Dictator Game, participants are given a sum of money and asked to decide how much to share with another individual, typically an anonymous recipient. In polarization research, the recipient’s partisan identity (e.g., Democrat or Republican) is subtly revealed. Iyengar and Westwood (2015) have found that participants allocate significantly less to out-party members, demonstrating that partisan bias contaminates even economic fairness judgments.

Similarly, the Trust Game is used to measure interpersonal trust and reciprocity. One player (the “trustor”) sends a portion of their money to another (the “trustee”), which is multiplied. The trustee then decides how much to return. When the trustee’s political affiliation is known, participants are less likely to trust or reciprocate with individuals from opposing parties. These findings show that polarization erodes basic social norms of cooperation and generosity, extending political conflict into domains unrelated to ideology or policy ( Westwood et al., 2018).

***The communication field***

In parallel, communication scholars investigate the role of emotional and moral language in media discourse and its effects on audience polarization. Brady et al. (2017) show that posts containing moral-emotional language, such as words evoking anger, disgust, or outrage tied to moral judgments (e.g., “corrupt,” “evil,” “betray”) are significantly more likely to be shared on social media platforms. These cues enhance in-group cohesion by reinforcing collective moral identity, while simultaneously heightening hostility toward out-groups, as messages are framed in stark moral dichotomies (good vs. evil). This dynamic of moral contagion helps explain why polarized narratives spread more rapidly and why they tend to entrench rather than bridge political divides.

Visual experiments investigate how imagery shapes political perception and emotional alignment, highlighting the symbolic and affective power of visual media in reinforcing or mitigating polarization. These studies assess how manipulated images, such as leader portrayals, national symbols, protest scenes, or digitally altered campaign visuals—influence audience judgments about trustworthiness, competence, moral character, or ideological alignment. For example, ( Zamora Medina et al., 2023) demonstrate how populist leaders utilize emotionally resonant imagery in conjunction with rhetorical appeals to portray themselves as the voice of “the people” against a corrupt elite, thereby fueling affective polarization by visually reinforcing in-group and out-group identities.

***Political science***

In experimental political science, research frequently examines elite cueing, which is the deliberate use of political messages by elites to guide partisans on how to interpret issues or events. Experiments expose participants to various statements from politicians or news sources to gauge their effects on attitude polarization, institutional trust, or democratic tolerance. This research has revealed that elite rhetoric significantly influences public attitudes toward democratic norms and institutions ( Wilson et al., 2020).

Specifically, when partisan leaders publicly undermine core democratic institutions, such as courts, elections, or the media, their followers are more likely to accept anti-democratic behavior. For example, Druckman, Peterson, and Slothuus (2013) find that when political elites frame issues in polarized, partisan terms, public opinion becomes more rigid, and support for institutional legitimacy wanes. Broockman, Kalla, and Westwood (2023) further demonstrate that even when affective polarization is reduced through interventions, elite-driven cues powerfully sway citizens’ willingness to endorse undemocratic actions, such as accepting election subversion or limiting checks and balances.

Similarly, Voelkel et al. (2023) show that depolarization interventions do not automatically enhance democratic resilience; individuals remain susceptible to anti-democratic attitudes when elite messaging erodes institutional trust. Cross-national experiments by Hameleers (2020) corroborate these findings, confirming that populist media messages, especially those depicting elites or institutions as corrupt, can diminish public trust in democratic governance across different contexts.

These studies collectively emphasize how elite rhetoric and partisan media exposure not only fuel polarization but also critically reshape civic norms, particularly when political messaging frames conflict in moral or identity-based terms. This body of research consistently highlights the causal impact of political communication in eroding democratic commitment, especially in highly polarized settings.

***Strengths and limitations of experimental methods***

Experimental studies are a central quantitative method in the study of political polarization, particularly within social and political psychology, behavioral economics, and communication research. They are widely used to test hypotheses, explore underlying mechanisms, and evaluate interventions aimed at reducing partisan hostility. Their major strength lies in their ability to establish causal inferences by controlling for confounding variables and isolating the effects of specific stimuli or treatments. For example, classic studies by Druckman et al. (2013) and Levendusky (2018) demonstrate how elite cues and identity priming (e.g., framing individuals as “Americans first”) can influence affective polarization and democratic attitudes. Experiments also allow researchers to evaluate interventions such as exposure to short videos about out-group heterogeneity, polarizing content warnings, or false belief correction tools, providing direct, data-driven insights into their effectiveness ( Voelkel et al., 2023).

The rise of internet-based experimental platforms, such as Prolific, MTurk, and Qualtrics, has made experimentation more cost-effective and scalable, enabling researchers to reach diverse populations and manipulate stimuli in controlled ways. Many experimental studies adhere to high methodological standards, including pre-registration, transparency in data and coding, and rigorous design protocols, enhancing their internal validity and replicability ( Lees & Cikara, 2020; Luttig, 2018). Moreover, these studies are often conducted in naturally polarized contexts, allowing researchers to examine how partisanship operates across different political identities (e.g., Democrats vs. Republicans) or even social groups (e.g., men vs. women). Behavioral economics complements this by using tools like the Dictator Game and Trust Game, revealing that partisan biases infiltrate apolitical domains such as resource sharing and interpersonal trust ( Iyengar & Westwood, 2015).

Despite these advantages, experimental studies also face several limitations. A central concern is their low ecological validity: findings derived from controlled environments do not always generalize to real-world, dynamic political contexts. Additionally, many studies rely on convenience samples rather than nationally representative populations, limiting their broader applicability ( Almagro, 2023). Polarization is also frequently measured indirectly through constructs such as affective polarization or group meta-perceptions, leading to variation in terminology and conceptual frameworks that complicate cross-study comparison. Philosophical critiques further question the authenticity of self-reported beliefs in experimental contexts, arguing that such expressions may reflect strategic identity signaling rather than deeply held attitudes ( Almagro, 2023; Hannon, 2021). Finally, much of the experimental literature remains geographically concentrated in Western democracies, limiting its global relevance ( Marino et al., 2024). While experimental research continues to evolve, with hybrid designs and cross-national replications helping to address some of these concerns challenges persist in ensuring that experimental insights translate into meaningful, context-sensitive understandings of political polarization.

### Computational and digital methods

As digital platforms increasingly mediate political life, the study of polarization has undergone a methodological transformation. Computational and digital methods now sit at the heart of contemporary polarization research, offering tools to capture and model the real-time dynamics of division across social, political, and cultural domains. From social media ecosystems to agent-based simulations, these approaches allow scholars to observe polarization as it unfolds, messy, multimodal, and emotionally charged in contrast to the static snapshots provided by traditional survey methods.

***Social media***

If internet panels bring scale, social media bring context—messy, dynamic, and emotionally loaded. Platforms like Twitter (now X), TikTok, and YouTube are no longer simply spaces where political discourse occurs; they are engines of polarization ( Frimer et al., 2022; Mueller & Sältzer, 2020). Through user-generated data, researchers can observe how identities form, narratives evolve, and emotions spread.

Early work on Twitter had revealed the presence of ideological echo chambers, challenging idealistic notions of digital platforms as new democratic forums. Users cluster by belief, algorithms filter content accordingly, and exposure to opposing views can paradoxically increase hostility rather than foster understanding ( Bruno et al., 2022; Weber et al., 2022). TikTok, in particular, adds a multimodal intensity, with sound, visuals, and text merging to deliver emotionally resonant political messaging. Kim et al. (2024) show how anti-vaccination content on TikTok leverages emotional storytelling to polarize audiences.

In response, researchers have tested interventions within the platforms themselves. From unfollowing hyperpartisan accounts to perspective-taking prompts, digital experiments now aim not only to study polarization but to disrupt it. Notably, interventions on Twitter by Rathje et al. (2023) showed a reduction in partisan animosity that lasted for over six months, demonstrating the real-world potential of platform-based behavioral nudges.

***Text, bots, and algorithms***

Computational tools such as sentiment analysis, emotion classification, and bot detection have become essential for mapping the affective architecture of polarization. Text analysis tools like VADER, LIWC, and BERTweet are widely used to extract moral-emotional language from tweets, Facebook posts, and YouTube comments.

Bot detection algorithms, such as Botometer, expose the inauthentic amplification of polarizing content. Linvill & Warren (2020) and Caldarelli et al. (2020) show how bots impersonating humans shift public perception, inflating the apparent support for extreme positions and spreading low-credibility information. These bots do not merely mimic opinion, they reshape the informational environment, and even minimal presence can shift average opinions in more polarized directions ( Pescetelli et al., 2022).

***Simulations and agent-based approaches***

To explore how micro-level behaviors translate into macro-level patterns of polarization, researchers have turned to computational simulations. Agent-Based Models (ABMs) allow for testing how social interactions, belief updating, or media exposure result in system-wide polarization ( Madden, 2025; Park et al., 2023). Törnberg (2021) modeled how algorithmic personalization and partisan sorting on digital platforms lead to entrenched affective divides, even in the absence of ideological extremism.

Similarly, Bayesian opinion dynamics models show that polarization can emerge even when individuals update beliefs rationally. Under certain conditions, such as selective exposure or noisy feedback—entire networks can drift apart, creating what philosophers call “epistemic bubbles” ( Pescetelli et al., 2022). These simulations complement observational data, providing a laboratory to explore “what-if” scenarios at scale.

***Visual and multimodal analysis***

While most digital research has focused on text, recent innovations have brought visual and multimodal content into analytic focus. Bast (2021)) and De-Lima-Santos et al. (2023) show how visual populism, from leader portraits to national symbols is crafted and circulated to elicit emotional allegiance and reinforce in-group identity. Platforms like Instagram and TikTok rely heavily on such cues, making computational visual analysis and semiotics essential tools.

Multimodal studies analyze images, soundtracks, memes, and symbols together to understand how polarization operates beyond words. Visual content is not only persuasive it’s performative, shaping how political groups see themselves and others. Image recognition algorithms and engagement metrics (e.g., on YouTube) are increasingly used to assess how audiences respond affectively to visualized politics ( Nordbrandt, 2023; Tran et al., 2022).

Digital ethnography and interpretive computational methods are increasingly used in tandem with machine learning techniques to contextualize algorithmically detected patterns. These hybrid approaches reflect the epistemological shift from purely predictive modeling to meaning-aware analysis rooted in media studies, digital sociology, and cultural semiotics. The rise of digital platforms has profoundly transformed the study of political polarization, with computational and digital methods now central to research. These advanced tools enable the capture and modeling of real-time dynamics of division across social, political, and cultural landscapes. Unlike traditional survey methods that offer static snapshots, approaches ranging from social media ecosystems to agent-based simulations allow scholars to observe polarization as a messy, multimodal, and emotionally charged phenomenon.

***Strengths and limitations of computational and digital methods***

Computational and digital methods have become essential tools in the study of political polarization, offering unique advantages over traditional quantitative methodologies. These approaches enable researchers to capture real-time, large-scale behavioral data across platforms such as Twitter/X, YouTube, TikTok, and Reddit, where polarization often unfolds in naturally occurring, emotionally charged contexts. Unlike laboratory experiments or survey-based studies, computational tools such as sentiment analysis, network modeling, and bot detection algorithms provide a window into how people express, share, and respond to political content at scale ( Bruno et al., 2022; Brady et al., 2017; Calderalli et al., 2020). This scalability allows researchers to identify emerging patterns, like echo chambers, ideological clustering, and moral-emotional contagion, that would otherwise remain hidden. Additionally, multimodal analysis enables the study of not just text, but also images, memes, sounds, and engagement behaviors, making these methods especially valuable on visual-first platforms like Instagram and TikTok ( Bast, 2021; De-Lima-Santos et al., 2023).

Another major strength of computational approaches lies in their ability to simulate complex polarization dynamics through agent-based models and Bayesian belief updating frameworks. These models allow researchers to test “what-if” scenarios, such as how algorithmic personalization or bot amplification might drive affective divides, even in the absence of ideological extremism ( Törnberg, 2022; Pescetelli et al., 2022). In parallel, digital platforms have become experimental spaces themselves, enabling researchers to embed and measure the effects of interventions in situ. For example, studies have tested the effects of unfollowing hyperpartisan accounts, exposure to cross-cutting perspectives, or the deployment of GPT-based AI bots that promote dialogue instead of division ( Rathje et al., 2023; Govers et al., 2024; Voelkel et al., 2024). These real-world interventions, measured over time and across user groups, reveal both the fragility and potential malleability of affective polarization in digital environments.

However, these strengths are tempered by important methodological and epistemological limitations. A key issue is context insensitivity: although text classifiers and sentiment models process language efficiently, they often miss cultural nuance, irony, or symbolic meaning, especially in multilingual or non-Western contexts. Many computational studies also rely on data from Western platforms and users, introducing geographic and demographic biases that limit the generalizability of findings ( Marino et al., 2024). Moreover, despite their sophisticated analytics, these methods frequently lack insight into belief authenticity; that is, whether user expressions reflect genuine positions or performative signaling tied to group identity ( Lees & Cikara, 2020; Almagro, 2023). This disconnect between observable behavior and internal cognition complicates both interpretation and causal inference.

Beyond methodological concerns, computational research also raises ethical and operational challenges. The scraping and analysis of user-generated content particularly without explicit consent pose privacy risks, while platform-specific dependencies make research vulnerable to algorithmic changes and data access restrictions. For example, shifts in Twitter’s API or Facebook’s content-ranking systems can disrupt longitudinal analyses and obscure replication ( De-Lima-Santos et al., 2023). Furthermore, the use of automated interventions, such as bot-driven moderation or algorithmic de-amplification, raises normative concerns about manipulation, bias, and democratic accountability. These limitations underscore the need for interdisciplinary approaches that combine the power of computational methods with ethical reflexivity, cultural sensitivity, and theoretical depth. As such, computational tools should be seen not as stand-alone solutions but as complements to interpretive and qualitative frameworks capable of unpacking the symbolic, affective, and institutional dimensions of polarization.

### Qualitative methods

Qualitative approaches offer deep insight into how polarization is experienced, narrated, and symbolically structured. These methods are particularly valuable for capturing affective, cultural, and discursive dimensions that may elude standardized survey instruments.

***Discourse and narrative analysis***

Drawing from linguistics, cultural sociology, and critical media studies, discourse analysis has become a central qualitative approach in polarization research, examining how political meanings are constructed, contested, and circulated. Through close analysis of speeches, social media posts, interviews, and news content, scholars trace how symbolic boundaries between “us” and “them” are discursively produced and sustained. Recent extensions of this approach, such as affective discourse analysis and multimodal critical discourse analysis (MCDA), have expanded its scope to include visual and emotive registers, particularly salient in polarized contexts like anti-vaccine activism, digital nationalism, and political crises ( Jaworsky, 2023; Yarchi et al., 2021).

Within this broader discursive tradition, narrative analysis plays a key role in uncovering the “deep stories” individuals tell to make sense of their political identities and perceived injustices. As Hochschild (2016) argues, these emotionally resonant accounts reflect not only competing ideologies but fundamentally different moral frameworks and emotional worldviews. Such narrative structures help explain why political polarization persists even in the face of factual correction: what is at stake is not merely information, but identity and meaning.

Scholars employ a range of discourse-analytic techniques to investigate how political antagonisms are reinforced symbolically and communicatively. For instance, MCDA is adept at unpacking the rhetorical and visual logic of internet memes and protest imagery, especially during moments of heightened political conflict ( Adebomi, 2024). Meanwhile, performative polarization, a concept developed by Revers (2023) is examined through systematic analyses of how political figures stage conflict across news and social media environments. This approach highlights how identity-based polarization is not only ideological but also performative and emotionally stylized, enacted through symbolic gestures, repeated scripts, and audience feedback loops.

In addition, researchers combine manual content analysis with automated text analysis to study polarization in large-scale digital environments. Techniques such as sentiment detection, misinformation tracking, and identity signal recognition are applied using tools like VADER and BERTweet, which process millions of social media posts to detect patterns of affective polarization ( Kim et al., 2024; Martin-Gutierrez et al., 2024; Rashid et al., 2024; Yarchi et al., 2021). These methods are used in conjunction with platform-specific analyses of user comments, on Facebook, Twitter/X, and WhatsApp to map how personal preferences become entrenched social identities within digitally mediated public spheres. Hence, discourse- and narrative-centered approaches provide essential interpretive depth to the study of polarization, capturing not just what people believe, but how those beliefs are shaped, felt, and defended in language and symbols.

***Ethnography and interpretive fieldwork***

Ethnography, rooted in anthropology and interpretive sociology, offers a powerful lens for studying political polarization as a lived, emotionally charged, and socially embedded experience. Rather than treating polarization as a purely cognitive or institutional phenomenon, ethnographic approaches emphasize how political divisions are experienced through embodied practices, everyday interactions, and identity performances. Anthropologists typically engage in long-term immersive fieldwork to uncover the cultural logics that underlie political attitudes and behaviors, while sociologists focus on how polarization is enacted through rituals, symbols, and public discourse ( Wedeen & Schatz, 2009). These methods bring to light the micro-level processes through which broader ideological cleavages are reproduced, resisted, or negotiated in daily life.

One key contribution of ethnography lies in its ability to uncover deep stories, emotionally resonant narratives that shape how individuals understand their social position and political belonging. Drawing from the sociology of emotions, Arlie Hochschild (1983, 2016) has pioneered the use of ethnography to explore how emotional attachments such as resentment, pride, or betrayal anchor political identities. Her work on conservatives in the U.S. South, for instance, illustrates how political beliefs are intertwined with moral frameworks and affective worldviews, often transcending factual disagreement. Ethnographic studies further show how political and non-political preferences are transformed into durable social identities, reinforced both in local interactions and across digital spaces ( Nordbrandt, 2023). Multi-sited ethnographies extend this analysis by tracing how global narratives such as populist conspiracies or diasporic grievances become embedded in specific cultural or geographic contexts, often mobilizing shared emotions like distrust or anger to foster collective identity.

Ethnographic and interview-based approaches are also crucial for exploring complex relationships between polarized actors and contested institutions, such as science. Rather than dismissing conspiracy theorists as irrational or “anti-science,” ethnographers investigate the ambivalent, context-dependent understandings people have of scientific authority and expertise ( Jaworsky, 2023). These methods reveal that mistrust in science is often not a blanket rejection, but a selective, emotionally mediated skepticism shaped by lived experience and perceived exclusion. Similarly, researchers apply unstructured ethnographic observation and discourse analysis to examine “performative polarization” the ways in which individuals and public figures engage in symbolic antagonism through media and everyday interactions, enacting political difference as part of their social identity ( Revers, 2023).

Finally, ethnography plays a foundational role in the sociology of emotions, where emotions are not viewed as irrational byproducts but as socially structured forms of knowledge. Influenced by concepts such as “affective economies” ( Ahmed, 2004), this tradition explores how emotions attach to political objects, circulate within groups, and generate solidarity or antagonism. Ethnographic fieldwork allows researchers to capture the emotional atmospheres surrounding polarized issues, observing how emotions like disgust, fear, grief, or indignation are produced and performed in situ ( Bericat, 2015; Collins, 1993; Salmela & von Scheve, 2017). This interpretive orientation aligns with structural hermeneutics in cultural sociology ( Alexander & Smith, 2003), which seeks to uncover the deep cultural codes and affective narratives that structure political conflict.

***Visual and multimodal analysis***

As political communication increasingly shifts to image-centric and emotionally resonant formats, Visual and Multimodal Analysis has emerged as a crucial qualitative methodology in the study of affective polarization. Particularly with the rise of social media, which thrives on multimodal content—combining visuals, audio, and text, this approach allows scholars to uncover how visual aesthetics, emotional cues, and symbolic representations shape political meaning and deepen partisan divisions ( Berrocal-Gonzalo et al., 2023; Kubin & von Sikorski, 2024).

Visual content, images, memes, and videos, plays an instrumental role in shaping public sentiment, reinforcing group identity, and mobilizing political emotions. Scholars have shown that political figures use visuals strategically to amplify affective appeals, often appealing to national identity, nostalgia, or culturally resonant symbols ( Joo & Steinert-Threlkeld, 2022; Kubin et al., 2021; Kubin & von Sikorski, 2024). On platforms like Instagram, visual storytelling becomes a central tool for crafting approachable or patriotic personas, as evidenced by Bast’s (2021) qualitative analysis of 724 Instagram images from right-wing populist politicians. Similarly, De-Lima-Santos et al. (2023) combine computational and qualitative analysis on over 11,000 images to track visual campaign trends across Brazilian presidential elections. These visuals often serve to polarize by intensifying emotional responses to political identity and opposition, especially when algorithmically amplified ( Yarchi et al., 2021; Kim et al., 2023).

Multimodal approaches extend this analysis by examining how text, sound, image, and gesture interact to construct emotionally persuasive narratives. Łabuz and Nehring (2024) highlight how the integration of music, meme aesthetics, and emotionally charged commentary contributes to the spread of polarized narratives. Research into anti-vaccination content on TikTok demonstrates that videos leveraging dramatic visuals and emotional storytelling elicit strong viewer reactions, thereby reinforcing in-group cohesion and antagonism toward out-groups ( Hunter, 2023; Kim et al., 2024). Adebomi (2024) employs multimodal critical discourse analysis to examine political memes and their role in pre-election affective crises, while Bouko et al. (2021) explore public sentiment during Brexit through shared Flickr imagery. These studies show that visual and multimodal communication does not merely reflect polarization; it actively performs and escalates it.

Recent work also investigates how visual cues can be used to detect and measure polarization. Joo and Steinert-Threlkeld (2022) review automated image analysis tools that assess how emotional and ideological content in visuals correlates with audience reactions. Peng and Lu (2023) demonstrate how comment sections on YouTube videos offer insight into online group polarization by analyzing engagement behaviors alongside visual narratives. These approaches enable a comprehensive mapping of both content and user responses, contributing to a deeper understanding of how visual environments foster polarized political ecosystems.

Importantly, researchers have begun to explore visual intervention strategies aimed at mitigating affective polarization. Tran et al. (2022), for instance, test the application of inoculation theory through “polarization content warnings,” proposing a system that flags emotionally manipulative content before users engage with it. This preventive strategy reflects a shift toward using visual design as a tool for emotional regulation and depolarization. While still emerging, these approaches suggest that visual literacy and content framing can play a role not only in understanding polarization, but also in counteracting it.

***Strengths and limitations of qualitative methods***

Qualitative methodologies are crucial for understanding the symbolic, emotional, and experiential facets of political polarization, which are often overlooked by quantitative analyses. Techniques like ethnography, discourse analysis, and visual/multimodal analysis enable researchers to explore how individuals develop meaning, political identities, and group boundaries through language, narratives, and emotions. For instance, Hochschild’s ethnographic research (1983, 2016) on “deep stories” illustrates how people construct moral worldviews and perceive injustice, demonstrating the role of emotions such as resentment and betrayal in shaping polarized political identities. Similarly, discourse analysts examine how polarization is actively performed through rhetoric, storytelling, and emotional framing in various media ( Revers, 2023; Jaworsky, 2023). These methods prioritize context, emotion, and narrative structure, providing a more nuanced comprehension of how political antagonism is felt and perpetuated in daily life.

Another advantage of qualitative methods lies in their capacity to capture multimodal and affective communication, especially as political discourse increasingly relies on visual and digital platforms. Visual and multimodal analysis reveals how images, symbols, and aesthetics are strategically used to evoke emotion and convey partisan meaning across platforms like TikTok, Instagram, and YouTube ( Kubin & Von Sikorski, 2024; De-Lima-Santos et al., 2023; Kim et al., 2023). These methods are uniquely suited to tracking the circulation of emotions, what ( Ahmed, 2004) terms “affective economies,” and how political meaning is constructed through visuals, sounds, and gestures. Interpretive methods also delve into identity formation, cultural scripts, and narrative coherence, offering a depth of explanation that complements the pattern-seeking nature of computational approaches.

However, qualitative methods do have notable limitations. A primary concern is their limited generalizability due to smaller sample sizes and context-specific findings, which can impede systematic comparisons across cases. While ethnographic immersion provides depth, it is time-consuming and often confined to specific locations or populations, raising concerns about external validity and selection bias ( Wedeen & Schatz, 2009). Critics also argue that qualitative research can be susceptible to interpretive subjectivity, particularly when the analytical framework is unclear or when researcher bias is not acknowledged. In visual and multimodal studies, interpreting imagery or emotional resonance remains analytically complex, and there is a risk of over-interpreting symbolic meaning without proper triangulation. Furthermore, while these methods excel at describing the experience of polarization, they are less adept at testing causal mechanisms or measuring the extent of attitudinal change. Consequently, qualitative approaches are most effective when combined with complementary methods, such as computational or quantitative approaches, to achieve a multi-layered, empirically grounded understanding of polarization in its symbolic, affective, and systemic forms.

## Concluding thoughts

In this review, we have provided an overview of the evolving study of political polarization. We have journeyed through different types of polarization and outlined the relevant concepts and theoretical approaches. Our central focus has been to elaborate the various methodologies and methods used to study political polarization, including quantitative, qualitative, and experimental methods, as well as the latest computational and digital approaches. We would like to conclude the review with some positive and hopeful suggestions, first offering some intervention strategies to encourage depolarization, and second, suggesting directions for future research, which involve cross-national approaches that extend to the global level.

### Intervention strategies and depolarization

In the existing literature, we have found various intervention strategies aimed at mitigating or “depolarizing” political polarization, acknowledging that depolarization is both a normative objective and a pragmatic necessity. The consensus across disciplines is that the toxicity of affective polarization, manifest in political animosity, social distrust, and democratic backsliding necessitates the systematic development of empirically tested interventions. Research on depolarization strategies is predominantly experimental in orientation, especially within psychology, political science, and behavioral economics. Interventions are grounded in theories of social identity, intergroup bias, epistemic accuracy, and civic engagement. Randomized Controlled Trials (RCTs) and online field experiments are common tools for assessing short-term and medium-term efficacy.

***Perspective-taking and empathy interventions.*** These strategies prompt participants to adopt the perspectives of ideological out-groups, aiming to humanize adversaries and reduce dehumanization. Voelkel et al. (2023) and Saveski et al. (2022) use large-scale online experiments to test the efficacy of guided empathy exercises. Broockman et al. (2023) and Pretus et al. (2024) employ behavioral experiments and economic games to measure downstream effects on trust and cooperation. While short-term reductions in partisan dislike are frequently observed, longitudinal persistence remains limited. Lees and Cikara (2020) note that corrective empathy may fade rapidly without institutional reinforcement.

***Priming shared identities.*** Drawing from social identity theory, these interventions emphasize supra-partisan identities (e.g., national, civic, or human identities) to reduce group salience. Levendusky (2018) have conducted priming experiments that frame participants as “Americans” first, reducing partisan hostility on affective and stereotyping measures. These methods are particularly effective in contexts with strong overarching identities but may backfire in fragmented or contested national settings.

***Correcting misperceptions.*** Misperception correction targets biased meta-perceptions, such as exaggerated beliefs about the extremity or hostility of the opposing side. Lees and Cikara (2021) show that providing participants with accurate data about out-group attitudes reduces affective polarization. However, the durability of these corrections is unclear, and some studies report backfire effects when participants perceive correction as partisan manipulation.

***Civic education and media literacy.*** Longer-term depolarization strategies focus on structural and cognitive resilience. Eroglu et al. (2024) implement a civic education and media literacy campaign across thirty-three countries, targeting young voters and media consumers. Their mixed-method evaluation indicates improved critical reasoning and reduced susceptibility to emotionally charged misinformation—factors that contribute to affective depolarization over time.

***Digital and social media interventions.*** Building on the understanding of how algorithms contribute to polarization, several studies have implemented in-situ interventions on platforms like Twitter. For instance, Saveski et al. (2022) encouraged users to interact with content that challenged their existing views. Similarly, Rathje et al. (2024) showed that unfollowing highly partisan accounts significantly and sustainably reduced affective polarization, with these behavioral changes lasting for six months. A new and innovative method being explored involves polarizing content warning systems ( Kubin & Von Sikorski, 2024). These visual interventions aim to mitigate the polarizing effects of news by applying inoculation theory, thereby strategically reducing polarization instead of exacerbating it.

***AI and bots for moderation.*** The common perception is that bots amplify political polarization. However, with the right application, they can actively reduce it. AI bots with specific mediation strategies can moderate online discussions by, for example, asking questions to encourage content generation instead of removing emotional posts. Govers et al. (2024) demonstrated this by using GPT-4-powered moderator bots in Reddit threads with varying discourse strategies. Bots that employed highly cooperative and consensus-framing approaches led to more civil discourse and decreased polarization among readers. This suggests that AI, when combined with deliberative design principles, can be a scalable intervention strategy.

***Understanding emotions as knowledge.*** From the sociology of emotions perspective, taking emotions seriously as a form of knowledge from both sides of a polarized debate can potentially contribute to depolarization and benefit democratic politics and deliberation processes. This approach acknowledges emotions as inherent in both affective and ideological polarization, and views emotions as a form of knowledge rather than a binary opposite to rationality. Equipping individuals with tools to manage their emotional responses to polarizing information, such as cognitive reappraisal or mindfulness interventions, can help them process information more constructively. As suggested by Bakker and Lelkes (2024), it gives individuals more control over the “informational” output of their emotions.

Recognizing that emotions are deeply tied to the narratives individuals adopt and the credence they give them, interventions can aim to alter these foundational stories or appeal to different forms of “knowledge” ( Almagro, 2023). One effective method is to present issues in ways that appeal to shared moral foundations or values that resonate across ideological divides. For instance, framing environmental conservation in terms of avoiding “pollution” or “contamination” can appeal to purity concerns, thereby increasing pro-environmental behavior across the ideological spectrum.

***Visual interventions.*** Visual interventions have emerged as an innovative strategy in the effort to counteract affective polarization, particularly within emotionally charged digital media environments. Recognizing the powerful role that images, symbols, and multimodal content play in shaping political perceptions and reinforcing partisan identities, researchers have begun to explore how visual design itself can serve as a tool for depolarization ( De-Lima-Santos et al., 2023; Joo & Steinert-Threlkeld, 2022; Kim et al., 2023; Łabuz & Nehring, 2024). These interventions aim to reduce intergroup animosity and foster cognitive openness by strategically altering the affective and interpretive conditions under which political content is consumed.

A prominent example of visual intervention is the development of polarizing content warning systems grounded in inoculation theory, a psychological framework that prepares individuals to resist persuasive or manipulative information. In a series of five experimental studies involving over 3,400 participants, Kubin and von Sikorski (2024) demonstrate that labeling political content as potentially polarizing prior to exposure significantly reduced expressions of partisan outrage and increased willingness to consider alternative viewpoints. These pre-emptive visual cues serve as subtle psychological prompts that mitigate the priming effects of emotionally provocative content, thereby promoting a more reflective mode of engagement.

Beyond warning labels, researchers are investigating how visual framing, through composition, symbolism, and affective tone can be intentionally crafted to foster common ground rather than amplify division. This includes efforts to depict political diversity without conflict, frame political issues around shared values, or use emotionally neutral imagery to dampen tribal reactions. Such framing strategies hold particular promise on platforms like TikTok, Instagram, and Facebook, in which multimodal content combining image, sound, and text heightens the emotional salience of political messaging ( Kim et al., 2023; Yarchi et al., 2021). While much of the existing visual political communication has historically reinforced in-group loyalty and out-group hostility, emerging research suggests that visual narratives can be designed to activate inclusive emotions such as empathy or shared vulnerability as a counterbalance to outrage and fear.

### Methodological considerations regarding intervention

Intervention research is predominantly experimental but increasingly interdisciplinary. Common outcome measures include affective thermometer scores, social distance scales, economic games (e.g., dictator or trust games), and engagement metrics on social media. Agent-based modeling has been used to simulate long-term effects of scaled interventions. The design and evaluation of intervention studies are shaped by several methodological strengths and persistent limitations:

• ***Ecological validity*:** Many laboratory-based studies lack contextual realism. Field experiments and in-platform interventions (e.g., on Twitter or Reddit) attempt to address this, but are often constrained by platform-specific dynamics or ethical limitations.
• ***Sample generalizability***: A large share of intervention studies relies on WEIRD (Western, Educated, Industrialized, Rich, and Democratic) populations, particularly U.S.-based online panels (e.g., MTurk, Prolific). This limits the external validity of findings across culturally diverse or global settings.
• ***Temporal durability*:** Most interventions assess short-term affective shifts, with few studies implementing longitudinal follow-ups. Exceptions (e.g., Rathje et al. 2024) demonstrate that behavioral effects can persist, but more studies are needed to confirm replicability and persistence over time.
• ***Ethical challenges*:** Interventions that leverage emotional triggers, identity cues, or algorithmic nudging raise normative questions about autonomy, manipulation, and unintended effects. Transparency, informed consent, and harm minimization are increasingly emphasized in experimental protocols.
• ***Measurement robustness***: Common tools such as feeling thermometers and stereotype indices provide accessible metrics, but they may reflect expressive responding rather than authentic belief change. Complementary use of behavioral and physiological measures (e.g., response time, gaze tracking) may offer richer insights.

### The broader context and theoretical insights on intervention

Beyond empirical design, disciplines such as cultural sociology, the sociology of emotions, and philosophy provide novel theoretical insights and critical frameworks for understanding the cultural and epistemic conditions of depolarization. For example, cultural sociology ( Morgan, 2022) proposes that populist grievances can be redirected toward “civil repair” if symbolic boundaries are reframed to emphasize universalistic civic values. This reframing involves discursive and performative shifts that transform exclusionary rhetoric into inclusive civic narratives, enabling marginalized groups to be re-incorporated into the civil sphere. The cultural mechanisms of meaning-making and symbolic boundary negotiation are central to these dynamics.

Scholars in working on the sociology of emotions argue that emotions are not simply irrational disruptions but are socially structured and politically meaningful ( Durnová, 2015). Treating emotional expressions, such as fear, anger, or resentment, as a form of knowledge opens new pathways for dialogical engagement and participatory reform. Emotional repertoires can be reconfigured to promote empathy, mutual recognition, and solidarity, particularly when institutions validate these emotions through inclusive public discourse.

Finally, philosophical analysis contributes uniquely to polarization research by offering tools for conceptual clarity, epistemic evaluation, and normative critique. Philosophers critically assess whether reported political attitudes, especially those captured through surveys, reflect genuine beliefs or merely expressive responses designed to signal identity or loyalty ( Kelly, 2008; Ridder, 2021). This distinction challenges the assumption that survey-based measures reliably capture internal cognitive states and calls for more refined interpretations of attitudinal data. Epistemic analyses examine the rationality and justification of belief formation processes in polarized contexts, probing how individuals update (or fail to update) their views in response to evidence and how group identity may shape the communicative intent behind political statements. In turn, normative inquiry addresses ethical questions concerning intervention strategies particularly whether they respect individual autonomy, avoid epistemic paternalism, and promote virtues such as open-mindedness, intellectual humility, and deliberative responsibility ( Marino & Iannelli, 2023; Durnová, 2015).

### Where do we go from here? cross-national perspectives on polarization

Recent scholarship increasingly emphasizes the comparative study of political polarization, particularly affective polarization, marking a conceptual and empirical shift away from the field’s earlier U.S.-centric focus. While foundational research has centered on ideological divergence within U.S. political elites and the electorate, contemporary studies underscore that affective polarization constitutes a global phenomenon. Yet, the causal mechanisms, affective repertoires, and behavioral expressions of polarization differ significantly across institutional, cultural, and historical contexts. This recognition is influenced, in part, by the expansion of social and political psychology, which often operates under assumptions of universal cognitive and emotional processes.

### Measuring affective polarization across contexts

To enable meaningful cross-national comparisons, scholars have developed and adapted quantitative indices. Reiljan (2020) has introduced the Index of Affective Polarization (API) based on Comparative Study of Electoral Systems (CSES) data, demonstrating that partisan animosity exists across both two-party and multiparty systems, including European democracies. Wagner (2021) proposes a complementary measure that calculates the dispersion of like-dislike scores toward political parties, weighted by vote shares. This approach allows for more granular differentiation across systems with varying party configurations. Researchers often rely on secondary data sources such as CSES, Eurobarometer, the European Social Survey, and national longitudinal panels to track macro-level trends and contextual drivers of polarization, including media structures, electoral systems, and political trust. While these indices have significantly advanced the empirical study of affective polarization beyond the U.S. context, their validity still depends on the comparability of cultural, institutional, and linguistic interpretations of survey items across countries.

### Cross-national research

A growing body of experimental research has extended the study of polarization across diverse national contexts. Westwood et al. (2018) employ behavioral experiments such as the Trust Game in multiple countries, revealing a robust correlation between affective polarization and reduced interpersonal trust. Gidron et al. (2022) have conducted a ten-wave longitudinal study in Israel, illustrating how affective polarization fluctuated in response to the COVID-19 crisis and its political handling. Harteveld et al. ( Harteveld, 2021; Harteveld et al., 2022) utilize multi-wave panel data across nine European countries to examine the effects of polarization on electoral participation and normative democratic attitudes. Comellas and Torcal (2023) have adapted Wagner’s metrics to analyze affective polarization in Latin American and Mediterranean democracies, affirming the broader applicability of this framework beyond Western Europe. Vanagt and Russo (2024) use survey data from 11 European countries to test the relationship between economic precarity and polarization, finding heterogeneous effects shaped by national welfare regimes. Neyazi et al. (2024) have implemented a survey experiment in India, demonstrating how exposure to sexist and uncivil political discourse modulates partisan hostility. Experimental interventions by Simonsson et al. (2021), Pretus et al. (2024), and Broockman et al. (2023) further illustrate the adaptability of experimental tools across political cultures and technological environments. Moreover, recognizing affective polarization as a transnational threat to democratic cohesion, scholars have begun evaluating mitigation strategies across national settings. Large-scale randomized experiments now test interventions such as empathy induction, identity reframing, and corrective information dissemination across diverse populations. Eroglu et al. (2024) have coordinated a civic education and media literacy intervention spanning thirty-three countries, with empirical evidence suggesting reduced susceptibility to polarizing narratives, particularly among younger, digitally immersed cohorts.

### Methodological challenges in comparative research

Despite the recent proliferation of cross-national research, several methodological constraints persist. Sampling bias is widespread, particularly in Global South contexts where non-probability online panels dominate. Geographic skew remains an issue, with a preponderance of studies focused on North America and Western Europe, limiting generalizability. Operational inconsistency in defining affective polarization impedes valid cross-case comparisons. Discrepancies in item wording, index construction, and cultural translations further complicate this issue. Nonetheless, interdisciplinary collaboration particularly across psychology, political science, and sociology, has facilitated the emergence of shared conceptual vocabularies and methodological innovations.

### Theoretical contributions to global understanding

Cross-national polarization research is significantly enriched by theoretical contributions from across the social sciences and humanities. In particular, cultural sociology, the sociology of emotions, and philosophical inquiry can lead us along a theoretical path toward a global understanding of polarization, intervention strategies, and depolarization. Cultural sociology, as articulated by Morgan (2022), theorizes how exclusionary populist rhetoric may be symbolically reframed to support processes of civil repair. This involves translating particularistic grievances into universal civic idioms that enable reintegration rather than antagonism. The sociology of emotions emphasizes that emotional repertoires are culturally situated. Contextual variation in what constitutes legitimate anger, resentment, or empathy has implications for how interventions are designed and interpreted across societies. Philosophical inquiry offers epistemic and normative tools for understanding belief polarization. By distinguishing between authentic belief and expressive responding, and by interrogating the ethical justification of political commitments, philosophy contributes to the critical assessment of both measurement validity and the goals of depolarization efforts.

The cross-national turn in polarization research reflects a maturation of the field. It combines empirical rigor with conceptual innovation, bridging diverse methodologies and cultural perspectives. Future research would benefit from further investment in comparative infrastructure, standardization of key constructs, and ethical reflection on the ends and means of political reconciliation across global contexts.

## Ethics and consent statement

Ethical approval and consent were not required for this article.

## Data Availability

No data were associated with this article.
