# Supplementary material for: The evolving study of political polarization
Source: Open Res Eur. 2026 Apr 7;6:93. [Version 1] doi: 10.12688/openreseurope.23252.1 (PMC13320125; doi:10.12688/openreseurope.23252.1)
Supplement: Peer review history

# Reviewer report: Reviewer response for version 1

**DOI:** 10.21956/openreseurope.25157.r75240

**Published:** 2026-06-30

**Authors:** Carol Galais, Fernando García

**Affiliations:** 1 Universitat Autònoma de Barcelona, Bellaterra, Spain; 2 Political Science and Public Law, Universitat Autonoma de Barcelona Departament de Ciencia Politica i Dret Public (Ringgold ID: 233407), Bellaterra, Catalonia, Spain

The manuscript addresses an ambitious and timely goal: providing an interdisciplinary overview of a rapidly expanding and increasingly fragmented literature on political polarization. This is undoubtedly one of its major strengths. The review moves beyond the traditional focus on ideological polarization, incorporating affective, identity-based, digital, and cross-national perspectives, and introduces readers to a broad range of disciplinary traditions (political science, social psychology, sociology, philosophy, and communication studies) while reviewing an equally diverse set of methodological approaches. Given the tendency for disciplinary literatures to develop in parallel rather than in dialogue, such an integrative effort is both valuable and necessary. The manuscript is clearly written, highly accessible, and remarkably up to date. For students and scholars entering the field, it is likely to become a useful point of reference.

 At the same time, our overall impression is that the review occasionally prioritizes breadth over analytical integration. Concepts, mechanisms, disciplinary traditions, methods, and empirical findings are brought forth, but the organizing logic connecting them is not always evident. As a result, the review sometimes reads more like a comprehensive inventory of the literature than an analytical map of the field. Many of the specific concerns raised below stem from this broader challenge.

**On the nature and transparency of the review**

 One initial question we found ourselves returning to: what kind of review is this, exactly? The manuscript presents itself as a comprehensive overview, yet the review strategy remains largely implicit. It is not clear whether the authors conceive of the manuscript as a narrative review, an integrative review, or a scoping review, each of which carries different expectations regarding literature selection, coverage criteria, and analytical ambition. The criteria guiding the selection of the literature are unspecified, and given the size of the field, readers would benefit from greater transparency on this point. The credibility and utility of any review rests substantially on its methodological explicitness. Stating clearly that this is an integrative review (or whatever the authors take it to be), and that literature selection prioritized breadth of disciplinary representation over exhaustiveness, would both strengthen the manuscript and set appropriate expectations for readers.

**On structure and organizing principles**

 More importantly, the manuscript would benefit from a stronger organizing principle both across and within disciplines. One option would be to structure each disciplinary section around a common set of dimensions: how polarization is conceptualized in that tradition, how it is measured, what methods are typically employed, what the major debates are, what causes and consequences have been identified, and what normative concerns arise. Such a parallel structure would make cross-disciplinary comparison considerably more tractable, and would help move the manuscript from a descriptive survey toward something more synthetic. Right now, some disciplines get more analytical depth than others. Social psychology and political science are treated at greater length and with more internal debate, whereas cultural sociology and the sociology of emotions feel somewhat thinner. Both, in fact, are quite questionable as “disciplines”. We discuss the case of emotions below. As for the Cultural Sociology section, we were occasionally unsure about the connection between some of the concepts discussed and the broader literature on political polarization. The paragraph devoted to Ernesto Laclau is a good example. While Laclau's work has been enormously influential in the study of populism, he is not generally regarded as a central contributor to the study of polarization. Introducing a discussion of populism at this point may therefore create a degree of conceptual distraction for readers unless the relationship between populism and polarization is explicitly developed. As currently written, the connection appears somewhat implicit, leaving readers uncertain about the specific contribution of this discussion to the study of polarization itself. In that sense, the paragraph on the authority of science could also be revised.

 Relatedly, many of the most consequential debates in polarization research appear only briefly or emerge indirectly. These include the elite versus mass polarization distinction, polarization versus partisan sorting (a debate the manuscript mentions between Fiorina et al. 2008 and Abramowitz & Saunders 2008, but does not really unpack), the symmetric versus asymmetric polarization debate, and the fundamental question of whether affective polarization is best understood as a cause or a consequence of ideological divergence. The normative debate is particularly important and largely absent as an explicit discussion. A great deal of contemporary polarization research is motivated by the concern that it contributes to democratic backsliding, declining institutional trust, and the erosion of deliberative norms, yet these concerns appear intermittently throughout the manuscript rather than as a coherent thread. Finkel et al.'s (2020) concept of "political sectarianism" captures this normative dimension well and would sit naturally in such a discussion. So does the growing literature questioning whether reducing affective polarization actually improves democratic attitudes. Broockman, Kalla & Westwood (2023) are cited, but the broader implications of their findings for the manuscript's own normative framing deserve more explicit treatment.

 A related structural concern is the treatment of depolarization research. The manuscript does include a dedicated section on intervention strategies, and it covers useful ground. But presenting depolarization as a concluding practical appendix, detached from the disciplinary and methodological sections that precede it, understates how theoretically loaded the question actually is. What counts as successful depolarization, and how it should be pursued, looks very different depending on whether one is working within a social-psychological, institutionalist, philosophical, or cultural-sociological framework. Integrating depolarization as a thread within each disciplinary section would make those differences visible and would strengthen the manuscript's integrative ambition. Namely, the contact hypothesis, with its well-documented effectiveness in reducing out-group hostility and discriminatory behavior (Scacco & Warren, 2018), could be introduced as a depolarization strategy alongside the more recently developed experimental interventions the manuscript already covers.

 The section's coverage also skews heavily toward experimental, individually targeted interventions (perspective-taking, identity priming, misperception correction), while structural and institutional approaches receive almost no attention. Electoral system design, public media regulation, and deliberative democratic institutions have all been proposed as mechanisms for managing polarization at the systemic level, but they are absent here, as well as the classic economic cleavages.

 Connected to this is a debate the manuscript touches on only briefly: the effects of affective polarization on civic engagement. This is one of the most contested and consequential questions in the current literature. Some research documents a mobilizing effect, where out-group hostility energizes electoral participation among those already politically active (Abramowitz & Webster 2016). Other work complicates this picture: Phillips (2024) shows that affective polarization mainly sustains existing habits of turnout without generating new participation, effectively exacerbating rather than correcting existing inequities in civic engagement. The systematic review by Lubej et al. (2025) maps this debate comprehensively and deserves more prominent treatment in the manuscript precisely here, as well as the meta-nalysis by Kołczyńska (2025).

 Finally, the philosophical account could be revised to make sure is parsimonious and discernable from empirical disciplines like psychology and the social sciences. If it involves interdisciplinarity (i.e., cognitive science), it should be addressed as such. The philosophical critique of experiments on page 17 is strong and could be connected to the concept of social desirability developed within social and political psychology.

**On the typology of polarization**

 The section on types of polarization is ambitious but conceptually uneven. The criteria used to define and distinguish categories are not always evident, and this matters, because without a clear classificatory principle, the typology risks generating more confusion than clarity. Indeed, false polarization looks less like a distinct type and more like a constitutive feature of polarized environments generally; similarly, interpretative polarization describes how polarization shapes cognition rather than what people are polarized about.

 Some categories clearly refer to forms or contents of polarization (ideological, partisan, nationalist). Others look more like mechanisms (interactional polarization), or cognitive features of polarized environments (false polarization, which is less a distinct type than a characteristic bias). Interpretative polarization, as defined in the manuscript, describes the epistemic process through which polarization shapes the reading of events, which is related to, but not the same as, the object of polarization. Horizontal and vertical polarization designate levels or directions of conflict rather than distinct types. Performative polarization, in Revers's sense, is perhaps better understood as a dynamic or mode of enactment than as a separate category.

 The manuscript would benefit from some explicit reflection on the logic of its own typology. Is it classifying by content (what people are polarized about), by target (toward whom), by mechanism (how polarization operates), or by level of analysis (elite, mass, interpersonal)? Bramson et al. (2017) offer a useful framework for precisely this kind of conceptual disambiguation, distinguishing several senses of polarization that are routinely conflated in empirical research, and their work would be a natural reference here. Additionally, Sunstein's work on group polarization (2002, 2009, 2017), the well-documented tendency for deliberation among like-minded individuals to push groups toward more extreme positions, is a notable omission. It is one of the most cited mechanisms in the literature and cuts across several of the types discussed in the manuscript. If fostering interdisciplinarity is part of the aims of the paper, the section could have a final paragraph reflecting critically on the over-typification both conceptually and methodologically. Such proliferation of categories risks producing fragmentation and insularity, ultimately weakening rather than enabling interdisciplinary work.

**On disciplinary architecture**

 We also found the disciplinary structure somewhat uneven. Some categories describe established disciplines (political science, social psychology), while others correspond more closely to theoretical traditions (sociology of emotions), explanatory mechanisms, or substantive domains (digital and visual communication). The boundary between political science and social psychology is particularly blurry here, given that social identity theory, which is foundational to both, sits at the centre of the affective polarization literature developed largely by researchers who publish in both fields. Iyengar and colleagues, for instance, are placed firmly in social psychology, when their institutional location and publication record put them closer to political science.

 For the psychology section, the link between stable personality traits and predisposition toward politically polarized behavior, beyond the psychometrically validated scales already covered, deserves more explicit discussion. Along the same lines, “Cool” and “warm’ feelings could be further explained for operationalization and conceptual clarity purposes.

 More substantively: while emotions are clearly central to contemporary polarization research, it is not entirely obvious that the "sociology of emotions" constitutes a distinct disciplinary section rather than a cross-cutting framework. Affective phenomena in polarization research are studied through affective intelligence theory (Marcus, Neuman & MacKuen 2000), cognitive appraisal approaches, intergroup emotions theory (Mackie, Devos & Smith 2000), narrative frameworks (Hochschild 2016), and computational sentiment analysis, and these come from very different disciplinary homes. Treating "sociology of emotions" as a discrete section risks underrepresenting the reach of emotional mechanisms across the whole manuscript. In sum, emotions are a frequent independent variable that many of the referred disciplines mention and develop from their own perspectives and using their own methods.

**On political science and affective polarization in comparative perspective**

 From the perspective of political science specifically, we were somewhat surprised by the limited attention devoted to the gradual shift from the study of elite polarization to mass polarization, a transition that has not only transformed the field's substantive focus but has profoundly altered its measurement strategies and methodological toolkit. Research on elites relies on legislative behavior, roll-call votes, party manifestos, and expert surveys. Research on citizens employs public opinion surveys, panel studies, survey experiments, list experiments, and increasingly social media data. This methodological bifurcation is consequential and deserved more systematic discussion, including its strengths and weaknesses.

 The treatment of affective polarization in multiparty systems is also less developed than one might expect in a 2026 review. The manuscript mentions Reiljan (2020) and Wagner (2021) but does not really engage with the substantial methodological difficulties of measuring affective polarization outside two-party systems: the aggregation problem, the asymmetry of inter-party affect, the distinction between hostility toward parties versus leaders versus ordinary voters (Kingzette 2021 is cited once and briefly), or the question of whether indices like Reiljan's Index of Affective Polarization actually capture the same construct as the feeling thermometer measures used in the US context. There is also a minor factual issue on this point: on page 7, the manuscript states that Reiljan's index integrates "leader aversion, symbolic antagonism, and moral superiority." This appears to be inaccurate. The API is based on weighted like-dislike evaluations of parties, not on those additional dimensions, and should be corrected.

 We would also highlight a tension that runs through the manuscript's treatment of digital environments. For a start, the concept of affordances is underutilized. This captures how the structural features of platforms shape what users can and are likely to do, and which has become a key analytical tool in social media research on polarization. Also, the manuscript treats echo chambers and algorithmic filtering as established mechanisms of polarization, but there is a growing body of evidence suggesting that echo chambers may be considerably less prevalent than commonly assumed (Eady et al. 2019; Arguedas et al. 2022; Budak et al. 2024). This does not mean platforms do not polarize, but the causal story is more contested than the manuscript implies. A more nuanced account of how social media algorithms manage visibility could help in that endeavor.

 Sometimes the concept “digital platforms” is coined when referring to social media (p.12; p.17): “The multimodal nature of digital platforms (…) intensifies the emotional salience of political messaging. (…) In contemporary digital environments”. For conceptual clarity purposes, we recommend avoiding using “digital platforms”, since it is particularly broad (Spotify, Apple music, Uber, and Amazon are also digital platforms). We recommend using “social media” or “social media platforms”. Along the same lines, it is not quite clear if the text claims algorithms do so as a result of LLMs or of this feature is just enhanced by LLMs (p.11). We would also suggest clarifying LLMs could amplify the already existing tendency of algorithms to favor polarization. Finally, the study by Tessler et al. (2024) could be included in the discussion on AI and bots.

**On methodologies and methods.**

 One of the more significant shortcomings in the manuscript concerns the methods section. Throughout it, the terms "methodology," "method," and "technique" are used more or less interchangeably, and this creates real analytical problems. The distinctions matter: quantitative research is a methodological orientation; a panel survey is a method; a feeling thermometer is a measurement technique. Experimental research is a design logic; a Dictator Game is a specific technique within it. Computational approaches constitute a broad methodological family; VADER or BERTweet are particular tools. Conflating these levels makes it harder to compare approaches meaningfully, and occasionally leads the section to treat things as equivalent that are not. A brief conceptual clarification at the outset  (even a short paragraph distinguishing methodology, method, and technique ) would considerably improve the section's analytical usefulness and set clearer expectations for what follows.

 A second, related issue is structural. Methodological choices are rarely made in isolation from theoretical concerns: experimental designs, surveys, computational tools, ethnography, and qualitative interviewing are typically selected because they address different questions, not because they are competing alternatives to one another. Presenting them as parallel options to be compared, each with its own generic list of strengths and limitations, can therefore give a slightly misleading picture of how research actually works. It also generates some repetition: discussions of digital communication, visual analysis, and computational approaches appear both in the disciplinary sections and again in the methods section, occasionally covering the same ground twice.

 One way to address both problems at once would be to integrate methodological discussions into the disciplinary sections themselves, showing how scholars from different traditions choose and adapt their tools in response to specific theoretical concerns. This would not only reduce duplication but would also make the manuscript's interdisciplinary ambition more visible at the level of structure, not just content. If the authors prefer to retain a standalone methods section, greater parallelism across subsections would help considerably: consistent headings, comparable levels of detail, and systematic attention to both strengths and limitations in each case would make the section much easier to navigate and more useful as a reference. For instance, Ahmed’s work on affective economies perfectly illustrates the case. Its inclusion is accurate and relevant, but it is worth mentioning that this perspective constitutes a theoretical approach that is better suited to qualitative study, but it is not reduced to ethnography. The study of the mechanisms by which emotions are attached to political objects could be conducted with almost any qualitative method.

**On geographical scope**

 The manuscript calls explicitly for expanding beyond the national and US-centric focus that has dominated the field, and we think this is exactly right. But the review itself does not fully deliver on this ambition. The section on cross-national perspectives mentions studies from Europe, Latin America, and India, but coverage of East Asia, Africa, and the Middle East remains thin, and the methodological challenges of comparative polarization research (construct equivalence across linguistic and political contexts, the limits of survey harmonization, the geographic biases of major datasets like CSES) receive only passing attention.

**A few additional points**

 The manuscript would also benefit from engaging more directly with the question of whether polarization is inherently dysfunctional. The review acknowledges, briefly, that some scholars argue strong disagreement may reflect legitimate mobilization of marginalized groups rather than democratic pathology, but this thread is not developed. It connects naturally to a broader debate about what kind of normative benchmark is implicit in polarization research: deliberative democracy, procedural democracy, stability-centered accounts?  

 On a related point, the literature on rational polarization deserves more explicit engagement than the manuscript currently offers. There is a compelling philosophical case, drawing partly on Kripke's dogmatism paradox and developed more recently by Dorst (2023) and Mercier (2020), that polarization can arise even among epistemically rational agents. If this is correct, the implications for intervention design are substantial: the mechanisms driving polarization may not be irrational biases to be corrected, but rather emergent properties of individually sensible belief-updating practices. Under this view, inoculation-style interventions risk addressing the wrong target entirely, since they assume a kind of epistemic malfunction that may not be present. This debate deserves at least a mention in the intervention section, where it would add important nuance to the otherwise largely optimistic account of depolarization strategies.

 Two further presentational points are worth raising. First, the final section currently contains four subheadings (Measuring affective polarization across contexts, Cross-national research, Methodological challenges in comparative research, and Theoretical contributions to global understanding) that would sit more naturally as subsections embedded within the existing "Where do we go from here?" section, rather than appearing as standalone headings of equivalent weight to the major sections preceding them. Their current formatting slightly overstates their structural independence from the cross-national discussion they are part of.

 Second, a minor but concrete gap: the passage on page 15 describing value affirmation as an intervention strategy ("Another strategy, value affirmation, prompts participants to reflect on core personal values, such as fairness or compassion. This approach has proven effective in reducing defensiveness when individuals encounter challenging viewpoints. Both identity priming and value affirmation aim to buffer individuals' identities, making them more receptive to opposing perspectives") is presented without any supporting citation. Given that the effectiveness claim is empirical and the rest of the interventions section is well-referenced, this should be addressed.

 More broadly, the concluding discussion feels somewhat summative where it might be more analytically ambitious. If one of the review's core aims is to foster genuine interdisciplinarity in the study of polarization, the conclusion is the natural place to make that case explicitly: to reflect on what the disciplines covered actually learn from one another, where their assumptions conflict rather than merely complement, and what an authentically integrated research agenda would look like in practice. As it stands, the conclusion largely restates what has been covered. A more critical and forward-looking close would better match the integrative ambition the manuscript sets for itself from the outset.

**Summary assessment**

 Overall, this manuscript has the potential to become a genuinely useful reference work for scholars of polarization. Its breadth, interdisciplinarity, and accessibility are real strengths, and the effort to bring disciplines into the same frame is valuable even when the integration remains partial. Our suggestions are intended not to reduce the manuscript's scope, but to help transform a broad survey of the literature into a more analytically integrated and structurally coherent contribution.

 References:

 Arguedas, A.R., Robertson, C.T., Fletcher, R. & Nielsen, R.K. (2022). Echo Chambers, Filter Bubbles, and Polarisation: A Literature Review. Reuters Institute for the Study of Journalism, University of Oxford.

 Hochschild, A.R. (2016). Strangers in Their Own Land: Anger and Mourning on the American Right. The New Press.

 Marcus, G.E., Neuman, W.R. & MacKuen, M. (2000). Affective Intelligence and Political Judgment. University of Chicago Press.

 Sunstein, C.R. (2009). Going to Extremes: How Like Minds Unite and Divide. Oxford University Press

Are all factual statements correct and adequately supported by citations?

Partly

Is the review written in accessible language?

Yes

Are the conclusions drawn appropriate in the context of the current research literature?

Yes

Is the topic of the review discussed comprehensively in the context of the current literature?

Yes

Reviewer Expertise:

Political science, public opinion

We confirm that we have read this submission and believe that we have an appropriate level of expertise to confirm that it is of an acceptable scientific standard, however we have significant reservations, as outlined above.

## References

[1] : Is Polarization a Myth?. *The Journal of Politics* .2008;70(2) : 10.1017/S0022381608080493 542-555 10.1017/S0022381608080493

[2] : Understanding Polarization: Meanings, Measures, and Model Evaluation. *Philosophy of Science* .2017;84(1) : 10.1086/688938 115-159 10.1086/688938

[3] : Does Affective Polarization Undermine Democratic Norms or Accountability? Maybe Not. *American Journal of Political Science* .2023;67(3) : 10.1111/ajps.12719 808-828 10.1111/ajps.12719

[4] : Misunderstanding the harms of online misinformation. *Nature* .2024;630(8015) : 10.1038/s41586-024-07417-w 45-53 10.1038/s41586-024-07417-w 38840013

[5] : Rational Polarization. *Philosophical Review* .2023;132(3) : 10.1215/00318108-10469499 355-458 10.1215/00318108-10469499

[6] : How Many People Live in Political Bubbles on Social Media? Evidence From Linked Survey and Twitter Data. *Sage Open* .2019;9(1) : 10.1177/2158244019832705 10.1177/2158244019832705

[7] : Political sectarianism in America. *Science* .2020;370(6516) : 10.1126/science.abe1715 533-536 10.1126/science.abe1715 33122374

[8] : Polarization in the American Public: Misconceptions and Misreadings. *The Journal of Politics* .2008;70(2) : 10.1017/S002238160808050X 556-560 10.1017/S002238160808050X

[9] : Does polarization increase participation? A systematic literature review and meta-analysis. *European Political Science Review* .2025;17(4) : 10.1017/S1755773925000116 553-568 10.1017/S1755773925000116

[10] : The Impact of Affective Polarization on Political Participation: A Systematic Review. *Political Studies* .2026;74(2) : 10.1177/00323217251338496 525-548 10.1177/00323217251338496

[11] : Intergroup emotions: Explaining offensive action tendencies in an intergroup context. *Journal of Personality and Social Psychology* .2000;79(4) : 10.1037/0022-3514.79.4.602 602-616 10.1037/0022-3514.79.4.602 11045741

[12] : Not Born Yesterday.2020; 10.1515/9780691198842 10.1515/9780691198842

[13] : Affective polarization and habits of political participation. *Electoral Studies* .2024;87: 10.1016/j.electstud.2023.102733 10.1016/j.electstud.2023.102733

[14] : Can Social Contact Reduce Prejudice and Discrimination? Evidence from a Field Experiment in Nigeria. *American Political Science Review* .2018;112(3) : 10.1017/S0003055418000151 654-677 10.1017/S0003055418000151

[15] : The Law of Group Polarization. *Journal of Political Philosophy* .2002;10(2) : 10.1111/1467-9760.00148 175-195 10.1111/1467-9760.00148

[16] : #Republic.2017; 10.1515/9781400884711 10.1515/9781400884711

[17] : AI can help humans find common ground in democratic deliberation. *Science* .2024;386(6719) : 10.1126/science.adq2852 10.1126/science.adq2852 39418380

# Reviewer report: Reviewer response for version 1

**DOI:** 10.21956/openreseurope.25157.r72121

**Published:** 2026-06-04

**Authors:** Manuel Almagro, Neftalí Villanueva

**Affiliations:** 1 University of Valencia, Valencia, Spain; 2 Philosophy I, University of Granada - Cartuja Campus (Ringgold ID: 83143), Granada, Andalusia, Spain

“The evolving study of polarization” offers an ambitious and much-needed interdisciplinary overview of the study of political polarization. More specifically, it helps organize a fragmented literature by distinguishing different types of polarization, surveying key disciplinary approaches, reviewing major methodological strategies, and identifying possible interventions and directions for future research. Its most original, and perhaps most important, contribution is that it brings together work from political science, social psychology, sociology, philosophy, communication studies, and computational social science, while rightly emphasizing that polarization is not a single phenomenon reducible to ideological distance. Polarization is a complex phenomenon, and for that reason its interdisciplinary study is especially important. In this respect, this overview is an extremely valuable contribution: it gives readers a broad and up-to-date picture of several central issues in the study of political polarization across different areas of inquiry. Anyone interested in understanding political polarization would certainly benefit from reading this review.

 We would like to contribute to complementing this interdisciplinary review. To this end, we offer a set of observations divided into two groups. First, we identify several issues that, in our view, could usefully be added to the review. Second, we offer a number of clarificatory comments that may help further refine some of the questions discussed in the article.

*Additional points*

 A discussion of the causes and consequences of political polarization could complement the material already included in this study, thereby distinguishing these issues more explicitly from questions concerning types, measures, and interventions. There is extensive work on both the causes and the consequences of polarization (see, for instance, Finkel et al. 2020; Kleinfeld 2023), and mentioning this literature would help provide a more complete picture of the different parts into which the study of political polarization can be divided across disciplines: types of polarization, causes, effects, measurement, and intervention.

 With respect to ideological polarization, two seminal contributions should also be added on board: Sartori’s (1976) work on party systems and DiMaggio, Evans, and Bryson’s (1996) work on attitude polarization. These are often cited as pioneering works on ideological polarization. Moreover, it would also be useful to mention Bramson et al. (2017), who distinguish several senses of ideological polarization that are often conflated, and therefore provide a highly relevant fine-grained conceptual approach to the phenomenon.

 Regarding the idea that echo chambers and algorithmic filtering are central mechanisms of polarization, it would be helpful to mention recent reviews suggesting that echo chambers may be less pervasive on social media than is often assumed (Arguedas et al., 2022; Budak et al., 2024; Eady et al., 2019; Fletcher et al., 2021, 2023; Tucker et al., 2022). There seems to be growing evidence pointing in this direction.

 One of the concepts with perhaps the most pedigree in the literature on polarization, missing in the overview, is “group polarization.” This does not refer to a further type of polarization, but rather to a mechanism, or family of mechanisms, that can contribute to increasing polarization. There is a very large body of work on this phenomenon, according to which discussion among like-minded individuals can lead groups to adopt more extreme positions (see Sunstein 2002, 2009, 2017).

 Another issue that has received increasing attention in recent years, both in philosophy and in other disciplines, concerns whether the mechanisms and processes that lead to political polarization are rational. Are polarized agents rational, or do people become polarized because they are insufficiently rational or cognitively sophisticated? Kevin Dorst’s (2023) and Hugo Mercier’s (2020) contributions are especially relevant to this debate. A further important philosophical contribution is Michael P. Lynch’s work on the kind of epistemic vice that may be connected to the rise of polarization (2020), as well as on the relation between truth, democracy, and polarization (2025).

 Some of these intuitions stem from a philosophical insight put forward by Saul Kripke in 1972 (Kripke 2011, 22 ff.) in the form of the so-called Dogmatism paradox. When confronted with new information, Kripke argued, the subjective probability assigned to the proposition presented as a new piece of information is modulated by the probability assigned to our previously held beliefs. When the new information is compatible with what we already believe, we assign to it a higher degree of subjective probability. When it goes against what we already believe, accepting it might take considerably more effort. There is a growing pressure to ignore it.

 There is nothing irrational about behaving in this way. We already assign high credence to our beliefs, especially when they form a web that we take to be consistent and suitably tied to reality. And it is perfectly rational to feel like the beliefs that we act upon are consistent with each other and true. As a matter of fact, it is conceptually impossible to consciously acknowledge that we entertain beliefs that are active and inconsistent with each other over a certain threshold of credence. Once we discover that some of our beliefs contradict each other, the rational thing to do is to get rid of the ones that seem most problematic. For similar reasons, it makes perfect sense to initially assign lower subjective probability to new information that contradicts what we think we already know.

 The problem this potentially poses for the ideal of democratic deliberation had already been explored by Kelly (2008) in one of the pioneering papers in the philosophical study of polarization. Disagreement does not necessarily lead to a moderation of our previously held views, or to a process of belief updating that leads us to abandon what we thought we knew as a consequence of the sheer force of the evidence presented by our interlocutors. Often, the effort devoted to the critical scrutiny of the evidence that contradicts what we thought we knew ends up contributing to the increase in subjective probability assigned to our previously held beliefs. What was supposed to make us more open minded and capable of appreciating the force of other people’s views, ends up contributing to the automatic rejection of those who are not like us.

 This point bridges the gap between the prevalence of echo chambers and rational polarization. It does not concern only the conceptual explanation of the phenomenon, though, it also affects the interventions that we imagine might ameliorate its problematic consequences. If echo chambers and the rise of polarization rationally arise from our well entrenched epistemic practices, it makes little sense to treat these phenomena as epistemic diseases, against which we can be inoculated. From an epistemological point of view, we are perfectly healthy even if we end up in an echo chamber, or with a disproportionate level of subjective probability assigned to the web of beliefs that we hold. Any intervention should keep in mind that we need to fight certain practices that usually work perfectly fine, and that most likely have provided an adaptive advantage in the past.

 A similar thought is expressed by Quassim Cassam when reflecting on the different dimensions of knowledge (Cassam 2019, 94). Cassam argues that our epistemic practices should be judged according to the extent to which they either facilitate or pose an obstacle to knowledge across three different dimensions: the acquisition, retention and transmission of knowledge. Our best strategy to acquire new knowledge is to be completely open to new evidence, even if it runs counter to our own beliefs. This attitude, however, would constitute a problem for the retention of the knowledge we already have and for the confidence needed to transmit what we know. If we are constantly questioning what we think we know, community practices and knowledge sharing might be seriously hindered.

*Clarificatory points*

 The paper moves quickly from ideological and affective polarization to a long list of “complementary and emerging forms” of polarization, including partisan, social, false, interpretative, interactional, horizontal, vertical, nationalist, and performative polarization. This is useful as a map of labels currently circulating in the literature, but it would also be useful to point out that most of them don’t pick out genuinely distinct types of polarization, in the sense that they don’t refer to different ways of conceiving public opinion division. For example, “horizontal” and “vertical” polarization are not straightforwardly additional types alongside ideological and affective polarization. In principle, both ideological and affective polarization can be horizontal or vertical (for a recent discussion, see Almagro 2025, ch. 2).

 When discussing affective polarization, it is assumed that standard measures capture people’s feelings and emotions. Yet it is not obvious why this should be so, or what exactly it means to measure “feelings” and “emotions” in the political context. At least some of the methods used to measure affective polarization may simply capture a form of political division that is independent of people’s *phenomenological* emotions. This is especially plausible for measures such as implicit association tests or behavioral games, which are aimed at attitudinal phenomena. For this reason, it would be useful to mention some recent work discussing what the “affective” in “affective polarization” amounts to. Relevant recent discussions include Röllicke’s (2023) work on the conceptual ambiguities of affective polarization in multiparty systems, especially concerning the object and nature of dislike; Bakker and Lelkes’s (2024) discussion of how affect is conceptualized and operationalized in affective polarization research; and the Handbook of Affective Polarization, edited by Torcal and Harteveld (2025), which includes dedicated chapters on conceptualization, measurement, causes, consequences, and interventions.

 Relatedly, it is important to note that the concept of affective polarization has recently been revised and reassessed (Almagro 2025). This contribution argues that it is a mistake to reduce affective polarization to its identity-based and emotional dimensions. More specifically, it claims that three further dimensions play a crucial role: the dimension of abstract discourse or narratives; the dimension of credence in certain ideas and narratives; and the dimension of emotional and non-emotional linguistic expression. The basic idea behind this alternative, revised conception of affective polarization is that affective polarization concerns the increase of affective, non-cognitive attitudes, such as credence, toward the core ideas of the political identity with which a group identifies, ideas that are typically articulated through a particular narrative. Under this conception, the “affective” in affective polarization is not boiled down to people’s emotions or phenomenal feelings, and the different measurement tools track information of different dimensions of one and the same phenomenon.

 With respect to the discussion of the potential benefits of polarization, it is important to note that both the possible democratic virtues and the disastrous consequences of increasing political polarization depend to a very large extent on the kind of polarization one has in mind. It makes little sense to ask about the benefits and harms of increased polarization in general without first clarifying what form of division in public opinion is at issue. A distribution of public opinion that simply contains a greater diversity of contents, or contents located closer to the extremes of a spectrum, will have neither the same potential benefits nor the same dangers as a distribution of public opinion in which, for example, one group has dramatically increased its affective confidence in the core ideas of the political identity with which it identifies.

 As noted above, one of the clearest lessons from the review is the recognition that the literature on polarization is complex and that we need a multidisciplinary approach if we are to properly understand the current state of the art. It remains an open question, though, whether we are discussing different features of a single phenomenon or placing genuinely different phenomena under the same umbrella term. In this respect, it is useful to go back to a basic question: why did we start worrying about polarization in the first place? Polarization is associated with a set of worries that concern the dynamics of public opinion. In particular, it is tied to a process that results in the partition of public opinion into groups that deny political legitimacy to each other to various degrees, in a context in which outliers are heavily penalized. This is not meant as an overarching characterization of polarization, rather, it is the acknowledgment that different kinds of polarization, whether or not they are tied to different disciplinary approaches and measurement techniques, are postulated to address a dynamic of public opinion that is increasingly incompatible with some central democratic ideals, such as democratic deliberation.

 Polarization is a multi-faceted phenomenon, one that we need to address through different disciplines, using different methodologies, to tackle both detection and intervention. But it is also necessary to keep in mind that detection and intervention should always aim at the amelioration of a distribution of public opinion that happens to be at odds with the democratic ideal of deliberation. In a society where public opinion is partitioned into groups that increasingly deny political legitimacy to one another, the open, honest discussion of our thoughts does not necessarily contribute to more inclusive decision-making practices, the kind that we respect for their intrinsic democratic value and for their essential contribution to the pursuit of truth.

**References**

 Arguedas, A. R., Robertson, C. T., Fletcher, R., & Nielsen, R. K. (2022). Echo chambers, filter bubbles, and polarisation: A literature review. https://ora.ox.ac.uk/objects/uuid:6e357e97-7b16-450a-a827-a92c93729a08/files/rzw12z639q

 Bakker, B. N., & Lelkes, Y. (2024). Putting the affect into affective polarisation. *Cognition and Emotion*, 38(4), 418–436. https://doi.org/10.1080/02699931.2024.2362366

 Bramson, A., Grim, P., Singer, D. J., Berger, W. J., Sack, G., Fisher, S., Flocken, C., & Holman, B. (2017). Understanding polarization: Meanings, measures, and model evaluation. *Philosophy of Science*, 84(1), 115–159. https://doi.org/10.1086/688938

 Budak, C., Nyhan, B., Rothschild, D. M., Thorson, E., & Watts, D. J. (2024). Misunderstanding the harms of online misinformation. *Nature*, 630(8015), 45–53. https://doi.org/10.1038/s41586-024-07417-w

 Cassam, Q. (2019). *Vices of the mind: From the intellectual to the political*. Oxford University Press.

 DiMaggio, P., Evans, J., & Bryson, B. (1996). Have Americans’ social attitudes become more polarized? *American Journal of Sociology*, 102(3), 690–755. https://doi.org/10.1086/230995

 Dorst, K. (2023). Rational polarization. *The Philosophical Review*, 132(3), 355–458. https://doi.org/10.1215/00318108-10469499

 Eady, G., Nagler, J., Guess, A., Zilinsky, J., & Tucker, J. A. (2019). How many people live in political bubbles on social media? Evidence from linked survey and Twitter data. SAGE Open, 9(1). https://doi.org/10.1177/2158244019832705

 Finkel, E. J., Bail, C. A., Cikara, M., Ditto, P. H., Iyengar, S., Klar, S., Mason, L., McGrath, M. C., Nyhan, B., Rand, D. G., Skitka, L. J., Tucker, J. A., Van Bavel, J. J., Wang, C. S., & Druckman, J. N. (2020). Political sectarianism in America. *Science*, 370(6516), 533–536. https://doi.org/10.1126/science.abe1715

 Fletcher, R., Robertson, C. T., & Nielsen, R. K. (2021). How many people live in politically partisan online news echo chambers in different countries? *Journal of Quantitative Description: Digital Media*, 1. https://doi.org/10.51685/jqd.2021.020

 Kleinfeld, R. (2023). Polarization, democracy, and political violence in the United States: What the research says. Carnegie Endowment for International Peace. https://carnegieendowment.org/research/2023/09/polarization-democracy-and-political-violence-in-the-united-states-what-the-research-says

 Kelly, T. (2008). Disagreement, dogmatism, and belief polarization. *Journal of Philosophy*, 105(10), 611–633.

 Kripke, S. A. (2011). On two paradoxes of knowledge. In Philosophical troubles: Collected papers, Volume 1 (pp. 27–51). Oxford University Press.

 Lynch, M. P. (2020). Polarization and the problem of spreading arrogance. In A. Tanesini & M. P. Lynch (Eds.), *Polarisation, arrogance, and dogmatism: Philosophical perspectives* (pp. 43–56). Routledge.

 Lynch, M. P. (2025). *On truth in politics: Why democracy demands it*. Princeton University Press.

 Mercier, H. (2020). *Not born yesterday: The science of who we trust and what we believe*. Princeton University Press.

 Röllicke, L. (2023). Polarisation, identity and affect: Conceptualising affective polarisation in multi-party systems. *Electoral Studies*, 85, Article 102655. https://doi.org/10.1016/j.electstud.2023.102655

 Sartori, G. (1976). *Parties and party systems: A framework for analysis*. Cambridge University Press.

 Sunstein, C. R. (2002). The law of group polarization. *The Journal of Political Philosophy*, 10(2), 175–195. https://doi.org/10.1111/1467-9760.00148

 Sunstein, C. R. (2009). *Going to extremes: How like minds unite and divide*. Oxford University Press.

 Sunstein, C. R. (2017). *#Republic: Divided democracy in the age of social media*. Princeton University Press.

 Torcal, M., & Harteveld, E. (Eds.). (2025). *Handbook of affective polarization*. Edward Elgar Publishing. https://doi.org/10.4337/9781035310586

Are all factual statements correct and adequately supported by citations?

Yes

Is the review written in accessible language?

Yes

Are the conclusions drawn appropriate in the context of the current research literature?

Yes

Is the topic of the review discussed comprehensively in the context of the current literature?

Yes

Reviewer Expertise:

Philosophy

We confirm that we have read this submission and believe that we have an appropriate level of expertise to confirm that it is of an acceptable scientific standard.

# Reviewer report: Reviewer response for version 1

**DOI:** 10.21956/openreseurope.25157.r72119

**Published:** 2026-06-02

**Authors:** Daniel Balinhas

**Affiliations:** 1 University of Limerick, Limerick, Ireland

Dear Authors,

 It has been a pleasure to read your paper “The Evolving Study of Political Polarization”. I have read it with great interest, and I do think that an integrative review like the one you have elaborated can be a great and necessary contribution to the interdisciplinary study of political polarization. In particular, this interdisciplinary approach is, in my view, one of the main strengths of the paper. I also really appreciate the effort that you have made to represent different disciplinary and methodological approaches, a task that is not easy at all due to the massive amount of articles on the topic, the messiness within the literatures dealing with polarization, along with the variety of approaches within polarization and intergroup conflict studies.

 For these two reasons, I do think that a potential second version of this paper addressing some aspects has high chances of being accepted without reservations. Having said that, I think that the reworking of the text should be notable, not so much by adding new elements (I think that the essential elements are already contained in the paper), but by reorganizing the review so that the contributions are clearer and readers can find it more useful and highly citable. The major points whose addressing would substantially improve the paper, in my view, are the following:

 1.    Clearly state the contribution and flag which type of review you are offering: I do agree that an interdisciplinary review is necessary, as you say in the second paragraph of the first section. However, I think that you somewhat understate your contribution, which in my view is not only “providing an overview”, but also putting into dialogue conceptualizations, normative assumptions, and empirical results produced within different disciplines that have not been in much dialogue with each other. Furthermore, in the section in which you address strengths and limitations of the different methodological approaches, you also offer critical insights and place an emphasis on the virtues of multi-method approaches. Thus, I would say that the typology of review you are offering is an “integrative review” or something similar. I think that explicitly stating what type of review you have conducted will help to: 1) orient readers so they know what to expect; 2) claim broader contributions; and 3) add more transparency, so readers understand your paper selection process, which is not exhaustive (thus, it is not a “systematic review”) but ensures a representation of different fields, which I would agree is the most important criterion for an integrative review or related types of reviews.

 2.    Types of political polarization: I think that the paper would enormously benefit from grouping different sub-types of polarization, as there are many different labels referring to very similar things. For instance, ideological polarization, issue-based polarization, left-right polarization, belief/value polarization, territorial polarization, etc. all have to do with positions towards different matters of public controversy. I do not see a strong reason not to include here what you call “nationalist polarization”, for example. This latter type of polarization, in my view, risks confusing more aspects than it clarifies, because if we talk about nationalist polarization, we could also talk about many other forms such as “populist” polarization, “anti-populist” polarization, “nativist” polarization, and so on. My recommendation would be to keep this classification as simple as possible, and provide integrative categories within which many sub-types of polarization can be included. 

 In a similar vein, the vertical/horizontal distinction and the parties/leaders/social groups distinction seem to have to do with who is the target of the (affective, cognitive, etc.) evaluation, so I think they could potentially be grouped together. Following this rationale, I think it would be very useful to provide readers with a table or graphical representation of the different polarization types. I know this is not an easy task, but I would say that this effort would translate into greater relevance and usefulness of your paper for readers trying to familiarize themselves with the polarization literatures. The same applies to the methods section, which could benefit from a clear table summarizing and systematizing the insights you provide.

 3.    “Methodologies and Methods” section: While I think that sub-sections by discipline work well in the first part of the review, I am less convinced that this works equally well for the section focused on methods. I do think that the different disciplines you mention probably contain a high degree of internal variability, both in terms of approaches and methods/methodologies. In this sense, I am somewhat reluctant to talk about “psychology methods”, “political science methods”, etc., as many social and behavioral sciences share the use of different methodologies and techniques. Also, while psychology does have an individual-analysis bias, many social and political psychologists (who I would say are among those most interested in polarization) promote other, less individualistic approaches. In addition, considerable sections of social psychology have historically provided theories and methods that clearly transcend a focus on individual-level analysis. Social Identity Theory and Social Representations Theory, both of which inspire contemporary social psychology, clearly transcend theoretical and methodological individualism. My example is about psychology because it is the field I am in, but this observation likely applies to all the disciplines you mention. In conclusion, I think that the way you structure the methodology section could perhaps break more clearly with disciplinary distinctions, something that I would say would be very consistent with the spirit of the paper. This does not mean that you cannot highlight that a particular methodology or approach has been more commonly used in X or Y discipline, of course. Also, I would recommend following a similar structure for quantitative, experimental, computational, and qualitative methods, as in this version the structure differs across sections.

 4.    Strengths and weaknesses of each methodological approach: Sometimes I feel that some of the strengths and weaknesses you mention could be applied more directly to the topic of political polarization, and not only inform the reader about limitations at a more abstract/general level (e.g., lack of ecological validity, lack of generalizability, etc.). Also, I would say that you could add more elements to some of the approaches you review. For instance, I would say that critical-qualitative methods and/or in-depth case studies within the polarization literature contribute by providing the necessary context, background, history of intergroup relations, and attention to power imbalances that help researchers avoid false equivalences. In this sense, qualitative methods can provide in-depth accounts of what particular groups are polarized about and what the nature of the political divisions they articulate actually is.

 Minor aspects:

 5.    I really like the different parts of the text in which you question the dichotomy between reason and emotion. I would personally love to read a somewhat expanded version of these sections, where you unpack why it would be productive to transcend this artificial distinction, for example, when dealing with affective polarization.

 6.    I would say that, at times, you locate particular scholars within disciplinary boundaries that might not be entirely accurate. For instance, Iyengar and colleagues have published more often in Political Science outlets than in psychology journals. Also, when you cite Abramowitz & Webster’s 2016 paper as among the first studies examining inter-party affect on page 5, I would say that the seminal paper of the “affective polarization literature” was probably Iyengar et al. (2012).

 7.    I am not sure that the “conceptual roots” of the study of polarization can be located only in Lipset and Rokkan’s work. There have been many insights within political philosophy, political science, history, economics, etc., throughout history dealing with political and societal conflict. Thus, I would say that the roots of polarization come from many different authors and intellectual traditions. I do agree, however, that cleavage theory provides a very insightful lens through which to think about polarization dynamics.

 8.    Checking some claims: On page 7, you write: “In multiparty systems, composite indices, such as Reiljan’s (2020) Index of Affective Polarization, integrate multiple dimensions, including leader aversion, symbolic antagonism, and moral superiority”. The way this sentence is phrased could give the impression that Reiljan’s index itself incorporates leader aversion, symbolic antagonism, and moral superiority, and I am not sure that this is the case.

 9.    Methods, methodologies, and techniques: I also think that it could perhaps be useful to provide a brief clarification or distinction between “methods”, “methodologies”, and perhaps even “techniques”, as these terms are sometimes used interchangeably throughout the paper. Given the interdisciplinary scope of the review, I believe that distinguishing more clearly between broader methodological orientations, concrete research methods, and specific research techniques could add further conceptual clarity for readers coming from different disciplinary traditions.

 It was a pleasure to review your paper, and I am looking forward to reading the second version. As you know, the review structure naturally makes us focus on aspects to improve, but I see many virtues and meaningful insights within the paper that will certainly enrich the political polarization literature. Please feel completely free to push back on any aspects I highlighted with which you disagree, and to engage in dialogue with me. I fully understand that not every point raised necessarily needs to be addressed in the exact way suggested here.

 Sincerely,

 Daniel Balinhas

Are all factual statements correct and adequately supported by citations?

Yes

Is the review written in accessible language?

Yes

Are the conclusions drawn appropriate in the context of the current research literature?

Yes

Is the topic of the review discussed comprehensively in the context of the current literature?

Partly

Reviewer Expertise:

Political Psychology

I confirm that I have read this submission and believe that I have an appropriate level of expertise to confirm that it is of an acceptable scientific standard, however I have significant reservations, as outlined above.
